# Characteristics of Potential Protein Nutraceuticals of Plant Origin with Antioxidant Activity

**DOI:** 10.3390/molecules25071621

**Published:** 2020-04-01

**Authors:** Iwona Szerszunowicz, Jan Kłobukowski

**Affiliations:** 1 Chair of Food Biochemistry, University of Warmia and Mazury in Olsztyn, Plac Cieszyński 1, Olsztyn-Kortowo 10-726, Poland; 2 Chair of Human Nutrition, University of Warmia and Mazury in Olsztyn, ul. Słoneczna 45f, Olsztyn-Kortowo 10-718, Poland; jan.klobukowski@uwm.edu.pl

**Keywords:** antioxidative nutraceuticals, BIOPEP-UWM database, *in silico* proteolysis

## Abstract

This study used selected plant proteins and the tools available in the BIOPEP-UWM database to profile proteins and release antioxidant nutraceuticals from their primary structures. The frequency of the occurrence of fragments with antioxidant activity in a protein sequence (the A parameter) was determined. A simulated monocatalytic proteolysis was carried out using ficin or stem bromelain or pepsin (pH > 2), and the theoretical degree of hydrolysis (DH_t_) and the frequency (including relative frequency) of the release of fragments with a particular antioxidant activity by a selected enzyme (the A_E_ and W parameters, respectively). Both barley hordoindolines and the protein group of “actins and other rice proteins” were characterised by the best antioxidant potential. On the other hand, among the main analysed cereal protein groups or species, the best nutraceutical sources included kafirins, rice glutelins and α-gliadins. Potentially the most nutraceutical molecules were released by pepsin (HL, VY, PHQ and PWQ biopeptides) from gliadins, but the most analysed proteins were hydrolysed (66% on average) and the DH_t_ for ficin and bromelain amounted to 27% and 31%, respectively. However, based on the calculated A_E_ mean values, it can be concluded that nutraceuticals were more frequently released from rice protein structures (IY and VY biopeptides), and less frequently released from barley and other cereal protein species, which may be of significance in the context of designing nutraceutical food.

## 1. Introduction

Nutraceuticals are bioactive compounds which are found in food and are isolated, biologically active ingredients that can be added to foodstuffs and food products. However, nutraceuticals also include dietary supplements (tablets, capsules). The consumption of such products and foodstuffs by humans results in greater health and therapeutic advantages compared to the consumption of conventional food [1,2,3]. Biologically active (bioactive) compounds include dietary fibre, oligosaccharides, polyunsaturated fatty acids (e.g., eicosapentaenoic acid (EPA), docosahexaenoic acid (DHA)), polyols (multi-hydroxyl alcohols), micro- and macronutrients, vitamins, lactic acid bacteria, choline and lecithin, and various phytochemical substances (phytocompounds) including polyphenolic compounds, plant pigments, herbs and their extracts [1,2,3]. These compounds contribute to delaying the body ageing process and extending the lifespan and prevent the development many chronic diseases including diabetes mellitus, hypertension, cardiovascular diseases (CVDs), Alzheimer’s disease (AD), Parkinson’s disease, eye disorders, allergies [2] and neoplastic diseases [2,4].

Certain nutraceuticals exhibit antioxidant activity and contribute to decreasing the levels of reactive oxygen species (ROS) formed in the body [4,5]. ROS play a significant role in the metabolism and the ageing of living organisms. They are formed under homeostatic conditions by mitochondria as by-products of intracellular metabolism but can also be exogenously induced by tobacco, drugs, contaminants, xenobiotics or radiation. These reactive forms of oxygen modulate various cell signalling pathways that are primarily mediated by transcription factors including nuclear factor kappa-light-chain-enhancer of activated B cells (NF-κB), signal transducer and activator of transcription 3 (STAT3), hypoxia-inducible factor-1, alpha subunit (HIF-1α) and growth factors as well as cytokines, enzymes and other proteins. Abnormal changes occurring in the cell, caused by ROS and oxidative stress, result in the development of many lifestyle diseases including neoplastic diseases in which neoplastic cells generate greater amounts of ROS [4].

The compounds that may have beneficial effects on the human body also include certain amino acids [3], peptides [5] and proteins [2,3], including enzymatic proteins [2,3,6,7,8]. Nutraceuticals are also biologically active peptides (BAPs), also referred to as biopeptides (BPs), which exhibit a broad spectrum of effects on the human body, including possible antioxidant activity [5,9,10,11,12,13,14]. They may be formed both under physiological conditions of the body, including in the human gastrointestinal tract under the influence of endogenous proteolytic enzymes, and also in food during various technological processes applied during food production and processing as well as fermentation processes [5,9,10,11]. Antioxidant peptides of food origin are regarded as safe and are characterised by high biological activity and are more readily absorbed by the body [5,13,14].

Proteins are a source of biopeptides of natural origin, including antioxidant biopeptides [5,9,10,11,12,13,14]. The amino acid composition of these biopolymers is determined by their type and origin of proteins (e.g., animal or plant). The essential amino acid composition in proteins is of particular importance in terms of nutrition and in the context of making use of this compound as well as its effects on the human body. Taking into account the amino acid composition of proteins, their bioassessment can be carried out using various criteria and indices. These indices include: Protein Efficiency Ratio (PER), Apparent and True Digestibility (AD, TD), Biological Value (BV), Net Protein Utilization (NPU), and the Protein Digestibility Corrected Amino Acids Score (PDCAAS). The latter was developed by the U.S. Food and Drug Administration (FDA) and the Food and Agricultural Organization of the United Nations and World Health Organization (FAO/WHO) as a modified form of the PER index, and since 2013, the Digestible Indispensable Amino Acid Score (DIAAS) has been used for the bioevaluation of protein [15,16,17,18,19].

Since various indices are applied for the biological evaluation of proteins and given the fact that each protein may be a source of biologically active peptides, as early as two decades ago, it was proposed to introduce a new criterion for the evaluation of protein as a source of biologically active peptides. Moreover, since computer methods and tools are increasingly applied in the processing of different biological data derived from various databases and are not only applied in molecular biology and genetics but also in nutrition and food sciences, the BIOPEP-UWM (Bioactive Peptides-University of Warmia and Mazury) database (http://www.uwm.edu.pl/biochemia/index.php/ pl/biopep) has been established [20,21]. Currently, one of the four databases which make up the resources of the BIOPEP-UWM database is the “Bioactive peptides” database in which 3,820 bioactive peptide sequences have been deposited (data as at 10 December 2019) in the form of a single-letter amino acid code. In addition it contains a program that enables the construction of profiles of potential biological activity of proteins as well as the calculation of qualitative and quantitative descriptors for the assessment of the value of proteins as potential precursors of bioactive peptides, and forecasting sensitive peptide bonds for the performance of protein chain hydrolysis by proteolytic enzymes [20,21]. Currently, the BIOPEP-UWM database is commonly used in food and nutrition sciences as a source of information on biopeptides as potential nutraceuticals and components of functional food as well as compounds that serve an important role in the prevention of chronic diseases [20,22,23,24]. Bioinformatic tools available in the BIOPEP-UWM database enable the performance of protein hydrolysis simulation with the use of proteolytic enzymes of various origins available in the database, including certain enzymes whose counterparts are synthesized in the human gastrointestinal tract or used in the food industry [8,20,25,26].

The study used selected plant protein amino acid sequences and bioinformatic tools available in the BIOPEP-UWM database to evaluate the potential antioxidant activity of the analysed proteins and a proteolysis simulation using selected enzymes to obtain potential nutraceuticals.

## 2. Results

Wheat proteins were the largest group of plant proteins subjected to bioinformatic analysis, which accounted for 45.7% of all analysed proteins and/or their fragments (Table 1). Due to their high number, i.e., 190 amino acid sequences and species diversity (*Triticum aestivum, Triticum aestivum subsp. Spelta, Triticum aestivum ssp. sphaerococcum, Triticum aestivum ssp. compactum, Triticum aestivum spp. tibeticum, Triticum turgidum subsp. durum*), the wheat proteins were divided into groups for comparison, both within the wheat protein group and with other analysed plant proteins (Table 1). Wheat proteins were classified into 11 groups, including α-, α/β-, γ-, ω-gliadins, glutenins (high molecular weight (HMW) or low molecular weight *(*LMW) subunits), enzyme inhibitors, thaumatin-like proteins, other proteins. Table 1 shows the number of amino acid sequences of proteins and/or wheat protein fragments available in the BIOPEP-UWM database resources, and the number and status of the studied sequences for which results were presented.

Bioinformatic analysis results were presented for these proteins and/or their fragments which satisfied the basic criterion for defining the molecules under study as biopolymers called proteins (a protein monomer needed to contain at least 100 amino acid residues). Each analysed protein was characterised in terms of the occurrence of potential sequence fragments with antioxidant activity (also referred to, in the study, as potential antioxidant sequence fragments or bioactive fragments which, after being released by proteolytic enzymes, are called antioxidant peptides or peptides with antioxidant activity).

The studied proteins were subjected to a specific “scanning” process which can be carried out in the BIOPEP-UWM database to find the so-called permutations with repetitions which indicate how many various fragments of sequences with antioxidant activity can be generated while having a monomeric protein molecule at one’s disposal (hence, *inter alia*, the term “potential”). To this end, the generated profile of potential antioxidant activity and its numerical equivalent, i.e., the A parameter, was used. Numerical values of the calculated A parameters, the number of potential bioactive fragments in the analysed protein sequence and the number of proteins in which such antioxidant fragments could be generated are presented in Table 1. Fragments of sequences with antioxidant activity are presented in the form of a single-letter amino acid code, in the same way as the fragments of amino acid sequences with different activities were recorded and are stored in the BIOPEP-UWM database resources.

### 2.1. Characteristics of Plant Proteins as Precursors of Bioactive Fragments with Antioxidant Activity

#### 2.1.1. Profiling Wheat Proteins in Terms of Antioxidant Activity

In the amino acid sequences of five different types of gliadins, composed of an average number of amino acid residues ranging from 265 (γ-gliadins) to 287 (α/β-gliadins) (Table 1), the most fragments with antioxidant activity were found in α-gliadins (an average of 14 bioactive fragments). For gliadin proteins belonging to five different analysed gliadin types, the average frequency of the occurrence of antioxidant fragments in a protein sequence (the A parameter) ranged from 0.0286 to 0.0498 (Table 1). The average value of the A parameter was the highest and amounted to 0.0498 for α-gliadins, which indicates that this protein sub-group may be a potential source (a better source) of amino acid sequence fragments with antioxidant activity, compared to other analysed gliadins.

In the analysed protein groups, the analysis results for the molecules recorded in the BIOPEP-UWM database resources with identification numbers (IDs) of 1435, 1436, 1437, 1438 and 1431 were not taken into account due to the adopted molecular criterion (at least 100 amino acid residues in a protein monomer). The molecules with the first two abovementioned IDs were composed of 54 amino acids and the next two had 15 amino acids, and the last had 24 amino acids. The results provided in Table 1 do not include the data originating from analyses of two other proteins either, even though they satisfied the conditions of the adopted molecular criterion. Gliadin proteins with IDs of 1365 and 1364 were composed of 120 and 146 amino acid residues, respectively. These proteins, however, were composed of a smaller number of amino acids compared to the analysed protein groups to which they had been classified, and, thus, the parameter A value calculated for them was very high and amounted to 0.1083 and 0.0959, respectively. The results for these two proteins are provided under Table 1.

In the primary structures (amino acid sequences) of gliadin proteins, the antioxidant fragments with the most potential were composed of 2-3 amino acids. These were primarily found in α-, α/β-gliadins. In these proteins, the most of such bioactive fragments which also contained a histidine molecule were the sequences LH, HL, AH and LHQ. Other dominant biosequences, but with a different amino acid composition were bioactive fragments LWQ, IR, TY, VY, LW, YQL, QYP, and in gliadins, a bioactive four-amino acid fragment of PYPQ was additionally found. Among the antioxidant fragments in γ-gliadins, bioactive four- and six-amino acid fragments were found, which occurred in more than 50% of the analysed proteins, and a rather unique bioactive fragment with a sequence of SVNVPL occurred but only in a small number of the analysed proteins (Table 1).

A rather high number of analysed proteins were glutenins, both HMW and LMW subunits (a total of 90 proteins). This group of proteins contained molecules composed of an average of 734 amino acids (HMW subunits) in which there were an average of 28 potential bioactive fragments and another group of proteins with an amino acid composition smaller and with a three times lower possibility of the occurrence of antioxidant fragments, i.e., glutenins, (HMW and LMW subunits), in them (Table 1). Unique motifs with antioxidant activity in the glutenin HMW subunit sequences were eight- and nine-amino acid fragments: LQPGQGQQ and LQPGQGQQG, respectively. However, in these subunits, a large number of potential antioxidant fragments with different structural motifs, i.e., the GYY, EL, RYY, WY, WYY, SYY, LK, PW, WG, were also observed. The average frequency of the occurrence of bioactive fragments in HMW subunits composed of a larger number of amino acids amounted to 0.0372, and for units with molecules smaller by more than four-fold, the calculated A parameter amounted to 0.0471 (Table 1). In other glutenin subunits, LMW, the most frequently noted structural motifs with antioxidant structure were the sequences LY, AH, IY, RHE, IQY, IR, VY, AY, PHQ, PWQ, PW, SVNVPLK, KP, HL and SHH (Table 1). In the analysed protein groups, similarly to the analysed gliadin proteins, the analysis results for the molecules with IDs of 1280, 1341 as well as with IDs of 1292, 1132, 1172, 1432, 1765 and 1770 were not taken into account due to the adopted molecular criterion (at least 100 amino acid residues in a protein monomer). These five latter molecules with the mentioned IDs were classified to a group of other proteins (Table 1).

Among the wheat proteins under study, enzyme inhibitors, thaumatin-like proteins and other proteins were analysed. The average value of the calculated A parameters for these components amounted to 0.0548, 0.0308 and 0.0474, respectively. The A parameter value for the analysed protein groups was the highest for the enzyme inhibitors. In the profiled proteins, two- and three-amino acid fragments with potential antioxidant activity occurred most frequently (Table 1).

#### 2.1.2. Profiling Rice Proteins in Terms of Antioxidant Activity

On average, from 144 to 875 amino acid molecules were found in subsequent plant protein monomers occurring in food and they were a large group of the analysed rise proteins (Table 2). Among these proteins, the smallest monomeric molecules were prolamins and globulins (an average of 144 amino acids per monomer), and the largest ones were enzymatic proteins and glutelins. Based on the calculated A parameters, it could be concluded that the best precursors of bioactive fragments with antioxidant activity were actins and other rice proteins (the A parameter = 0.0734), enzymatic proteins and glutelins, oryzains and allergenic proteins (the A parameter range of 0.0551–0.0566). In these proteins, a large number of bioactive antioxidant fragments with a diverse amino acid composition were found. Enzymatic proteins and other proteins that serve significant biological functions at the cellular level in rice grains were characterised by a rich profile of antioxidant activity (Table 2). Among the antioxidant sequence fragments, the dominant ones were fragments with the molecules of histidine (HH, HL, LH, LHL, RHD), alanine (AH, AW, AY), leucine (EL, IKL, LK, LY, LW, LH, LHL), lysine (KD, KP, VKP), arginine (IR, RW), tyrosine (AY, IY, VY, LY, TY), tryptophan (AW, RW, WG, VW) and the FC, MY and MM. In rice prolamins (and globulins), the frequently occurring bioactive fragments were the sequences of VW, IY, YQL, WY and MM, and in glutelins, the sequences of AH, EL, IR, LK, TY, FC, PWH, PW and KP while in both group of the proteins concerned, the most frequently occurring amino acid sequences were AY, LY, IY, VY, HL (Table 2).

In the group of analysed glutelins, nine molecules (IDs of 1582-1590) in which the number of amino acid residues in the polypeptide chain was lower than 100, were not taken into account. In groups of other rice proteins, compounds with IDs of 1553, 1707 and 1710 were also disregarded.

#### 2.1.3. Profiling Barley and Oat Proteins in Terms of Antioxidant Activity

The studied monomeric sequences that were used in bioinformatic analyses also included barley proteins (59 sequences) and oat proteins (23 sequences) (Table 3 and Table 4). Similar to wheat and rice proteins, in these two groups of analysed proteins presented in Table 3 and Table 4, the study results obtained for barley molecules, including five compounds classified as hordeins (IDs 1606, 1607, 1624, 1629 and 1630) and four other barley proteins (IDs 1612, 1620, 1656 and 1175), were not taken into account. Moreover, the results of analyses of oat amino acid molecules belonging to avenins (IDs 1450, 1459, 1460, 1461) and other proteins (IDs 1447-1449) were also not taken into account.

In amino acid sequences of six various analysed groups of barley proteins composed of an average ranging from 136 (hordoindolines) to 450 (tubulin chains) amino acid residues, most bioactive antioxidant fragments were found in tubulin chains (an average of 16 bioactive fragments), and in hordeins and globulin, hordothionins and other barley proteins (an average of 13 bioactive fragments, Table 3). The average calculated value of the A parameter was the highest for hordoindolines and globulin and hordothionins, and for enzymatic proteins and other proteins. The calculated average values of the A parameter amounted to 0.0773, 0.0587 and 0.0530, respectively (Table 3). On the other hand, the smallest studied oat molecules (up to 169 amino acid residues) were the proteins classified to the group of proteins–avenoindolines and other oat proteins, and thaumatin-like pathogenesis-related proteins, for which the calculated average values of the A parameter were the highest and amounted to 0.0642 and 0.0488, respectively (Table 4). Of all the analysed oat proteins, the most antioxidant sequence fragments with a diverse amino acid composition were found in oat globulins. In these proteins, bioactive fragments of the sequences AY, LY, AH, IR, LKP, LK, KP, TY as well as PWQ, KD, PW, VY and FC were dominant (Table 4).

#### 2.1.4. Profiling Buckwheat, Rye and Sorghum Proteins in Terms of Antioxidant Activity

Buckwheat proteins were represented by globulins, legumin-type proteins, vicilin-like protein and other proteins which, in total, accounted for 13 analysed amino acid sequences (Table 5). The bioinformatic study also used three sorghum monomers and three rye monomers, while excluding the results of analyses on compounds with IDs of 1439 and 1443-46, referred to as secalin fragments, and fragments of other rye proteins (IDs 1440-42, 1594, 1596-98, 1601).

Among the studied buckwheat, rye and sorghum proteins, the highest values of the calculated A parameters were for kafirins, i.e., sorghum proteins (an average A value of 0.0523), buckwheat proteins i.e., globulins (an average A value = 0.0460), and the group of allergenic proteins and other buckwheat proteins (an average A value = 0.0420) (Table 5). In the amino acid sequences of buckwheat globulins, most potential antioxidant fragments were composed of 2-3 amino acids, and the dominant bioactive fragments were the sequences HH, LY, AH, EL, RWN, PW, RW, IR, LK, FC, AY (Table 5). However, the unique motifs with antioxidant activity in the rye glutenin HMW subunit sequences were the oligofragments LQPGQGQQ and LQPGQGQQG. In these subunits, however, a large number of potential antioxidant fragments with different structural motifs were observed, i.e., the GYY, EL, EYY, YYL, YYI, SYY, LK, KP, WG (Table 5).

### 2.2. In Silico Hydrolysis of Selected Plant Proteins with the use of Selected Enzymes (BIOPEP-UWM)

1248 simulations of protein molecule proteolysis were carried out using the bioinformatic tools (*in silico* protein hydrolysis) available in the BIOPEP-UWM database. Enzymatic monocatalyses were carried out using ficin (EC 3.4.22.3) or stem bromelain (EC 3.4.22.32) or pepsin (pH > 2) (EC 3.4.23.1) and a bioevaluation of the conducted processes was then carried out. The bioevaluation was conducted based on the obtained data on the bioactivity of the obtained proteolysis products (the number, the type of peptides with a given activity, including peptides with antioxidant activity), and the bioparameters characterising the process were calculated (DH_t_, A_E_, W) (the parameters were characterised in detail in the “Materials and Methods” section of the study).

#### 2.2.1. Bioevaluation of Wheat Protein Proteolysis Simulation Process and Products

Only certain monomers (i.e., α-, α/β-, ω-gliadins) were hydrolysed by ficin in an amount of approx. 24–28%, and, potentially, the enzyme could release, from each of these protein molecules, one antioxidant peptide or three biopeptides (only for one ω-gliadin out of the three analysed proteins), even though no biopeptide with the preferred activity was obtained from some sequences of γ-gliadins (Table 6). By carrying out the hydrolysis process using bromelain, an average of one antioxidant peptide could be obtained from almost all γ-gliadins subjected to hydrolysis, and one biopeptide from each of the remaining gliadin monomers. An average range of the DH_t_ calculated for the process using bromelain, with gliadins as substrates, amounted to 26–34% (Table 6). Of the three enzymes used for the proteolysis simulation, pepsin (pH > 2) exhibited the highest effectiveness in hydrolysing peptide bonds found in gliadin molecules, while releasing from these proteins from one to three peptides with antioxidant activity (an average range of the DH_t_ amounted to 64–67%) (Table 6). Irrespective of the proteolytic enzyme used to carry out a gliadin hydrolysis simulation, α- and γ-gliadins can be a better source for obtaining antioxidant peptides. The average value of the calculated bioparameter A_E_ for α- and γ-gliadins amounted to 0.0044 and 0.0041, respectively (for the process carried out by ficin), and 0.0042 and 0.0045 (for the process carried out by bromelain), and 0.0064 and 0.0090 for pepsin (pH > 2). On the other hand, the average value of bioparameter W amounted to 0.0907 and 0.1231, respectively, 0.0853 and 0.1452, and 0.1285 and 0.2666 (Table 6). Most antioxidant peptides with the TY sequences were obtained from gliadin proteins as a result of using ficin to carry out the hydrolysis.

**Figure 1:**
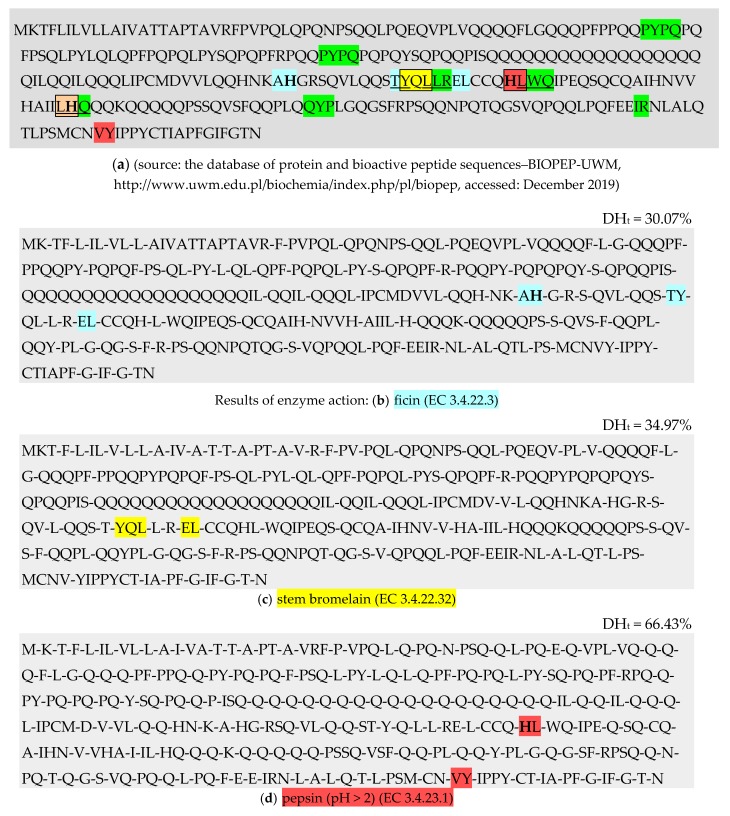
The amino acid sequence of α-gliadin (wheat, *Triticum aestivum*, ID 1304) containing selected fragments with antioxidative activity.

On the other hand, bromelain released more bioactive sequences YQL and IR from the analysed protein groups, and among the bioactive products obtained as a result of applying pepsin (pH > 2), there were most biopeptides with sequences HL, PHQ and VY (Table 6, Figure 1).

Glutenin proteins (subunits HMW and LMW), similar to gliadin monomers, were hydrolysed to the greatest extent by pepsin (pH > 2) (an average DH_t_ value for all analysed glutenins amounted to 67–75%), and bromelain hydrolysed peptide bonds in two glutenin groups (subunits HMW) in an average of 41% and 50% (Table 6), releasing up to 3-4 antioxidant peptides. High bioparameter values (A_E_ = 0.0156, W = 0.3213) and (A_E_ = 0.01474, W = 0.2976) were obtained as a result of using ficin or bromelain to carry out the hydrolysis of one of the two analysed groups of glutenin proteins WG and EL are sequences of the antioxidant peptides most frequently released by these two enzymes (Table 6).

#### 2.2.2. Bioevaluation of the Rice Protein Proteolysis Simulation Process and Products

Under conditions of simulated rice prolamin and globulin hydrolysis by ficin, approx. 41% of peptide bonds were degraded by the enzyme (Table 7). The frequency and relative frequency of the bioactive antioxidant fragments being released by ficin from prolamins and globulins (A_E_ and W parameters) amounted to 0.0115 and 0.2389, respectively. The numerical value of the A_E_ parameter was comparable to the processes carried out when using bromelain or pepsin (pH > 2), even though the enzymes hydrolysed monomeric prolamins and globulins to varying degrees. An average DH_t_ amounted to 54 and 70%, respectively for the processes in which rice prolamins and globulins were substrates, and bromelain and pepsin (pH > 2) were the enzymes used to carry out the hydrolysis simulation. Although glutelins were enzymatically degraded by ficin or bromelain or pepsin (pH > 2) to a similar degree, the latter enzyme released from the studied proteins more antioxidant peptides (the A_E_ parameter = 0.0106, W parameter = 0.1916) with a more diverse peptide profile. Most antioxidant peptides with sequences of IY or VY were released from prolamins and globulins as well as from glutelins (Table 7).

Among the products obtained from enzymatic degradation (using ficin or pepsin (pH > 2)) of allergenic and enzymatic proteins, there were antioxidant peptides with a very rich and diverse peptide profile, obtained particularly after the degradation of enzymatic proteins. The calculated average value of the A_E_ and W bioparameters amounted to 0.0135 and 0.2934, respectively, for the process carried out by ficin, and to 0.0126 and 0.2520 for the simulated hydrolysis of allergenic proteins, during which pepsin (pH > 2) was used (Table 7).

#### 2.2.3. Bioevaluation of the Barley and Oat Protein Proteolysis Simulation Process and Products

During the simulated barley protein hydrolysis for which ficin was used, most antioxidant peptides were released from tubulin chains, and from the enzymatic protein group as well as hordothionins (Table 8). For these proteins, the calculated average value of the A_E_ parameter amounted to 0.0106, 0.0149 and 0.0188, respectively, and of the W bioparameter, to 0.3082, 0.2782 and 0.2383, respectively. Most antioxidant peptides were released from tubulin chains (sequences of AY, WY, MY, IR, TY) and from hordothionins (most with the sequences of AY, EL, TY and WG). Ficin released from hordeins an antioxidant oligopeptide with a sequence of PQIPEQF (Table 8).

Bromelain degraded hordothionins the most (an average DH_t_ of approx. 56%) while releasing biopeptides with sequences of HL, IR, EL, WG and PWG. Average values of the calculated A_E_ and W bioparameters amounted to 0.0113 and 0.1416, respectively, and while the value calculated for the A_E_ bioparameters was the highest compared to other analysed barley protein groups, the value of the W bioparameter was the highest for hordeins (Table 8). The conducted *in silico* hydrolysis of the same barley protein group using pepsin (pH > 2) yielded hydrolysates with a structurally diverse profile of antioxidant peptides, and the calculated A_E_ and W bioparameters amounted to 0.0171 and 0.2713, respectively. In hydrolysates of all analysed barley protein groups obtained from the conducted proteolysis simulation using bromelain, the IR and EL biopeptides were found. Pepsin (pH > 2) did not release any antioxidant peptide from hordoindolines, and in hydrolysates of other barley proteins, the peptide with a sequence of VY was the recurrent biopeptide (Table 8).

During the hydrolysis of the thaumatin-like pathogenesis-related proteins classified to oat proteins, simulated by ficin and bromelain, no peptide with antioxidant activity was obtained (Table 9). Most antioxidant fragments of amino acid sequences were released from oat globulins by bromelain. Bromelain and pepsin (pH > 2) hydrolysed 51% and 71%, respectively, of all available peptide bonds in these protein molecules, and most biopeptides were found among the products obtained due to the use of the specificity of pepsin (pH > 2) action (Table 9).

#### 2.2.4. Bioevaluation of the Buckwheat, Rye and Sorghum Protein Proteolysis Simulation Process and Products

Hydrolysing certain buckwheat proteins: legumin-type proteins, vicilin-like protein and rye proteins (secalins) by using pepsin (pH > 2) to this end yielded no bioactive peptide (Table 10). Most antioxidant peptides were released from buckwheat globulins by bromelain or pepsin (pH > 2), and the average value of the A_E_ bioparameter was 0.0053 irrespective of the enzyme used while the average value of the second bioparameter (the W parameter) amounted to 0.1216 and 0.1157, respectively, subsequently for the proteins hydrolysed by bromelain or pepsin (pH > 2). The theoretical degree of hydrolysis amounted to 55% (for the process carried out by bromelain) and 68% (for the process carried out by pepsin (pH > 2)). Among buckwheat globulin hydrolysis products obtained due to bromelain action, the most frequently released antioxidant peptides included EL and IR peptides (Table 10).

## 3. Discussion

### 3.1. Plant Proteins as Precursors of Bioactive Fragments with Antioxidant Activity

Proteins are amino acid biopolymers that serve various functions in the human body and are among the main nutrients for the bioevaluation for which various indices, including PER, AD, TD, NPU, PDCAAS, DIAAS [15,16,17,18,19], are used. These biopolymers are a source of peptides with a broad spectrum of biological impact on the human body [5,9,10,11,12,13,14,27,28,29,30]. BAPs precursors can also include wheat, rice, barley, rye, oat and sorghum proteins (Table 1, Table 2, Table 3, Table 4 and Table 5) [27,28,29,30].

Cereals are a staple food for people in many regions worldwide, and their carbohydrate content allows them to satisfy approx. 50% of daily human energy needs [30]. Cereal grains also contain proteins whose contents account for 6–20% of the grains [30,31,32,33]. These include albumins (soluble in water), globulins (soluble in salt solutions), prolamins (soluble in alcoholic solutions) and glutelins (soluble in acids or bases) [33,34]. The dominant protein groups found in cereal grains are prolamins and globulins. Prolamins found in wheat, rye, barley and sorghum account for 30–50% of all proteins and, depending on the type of cereal in which they occur, are referred to as gliadins, secalins, hordeins and kafirins, respectively [30,34,35,36]. In oat and rice grains, prolamins account for approx. 4-15% [30,31,32]. Cereal proteins are characterised by a lower nutritional value compared to other food proteins. In protein structures, there are few molecules of lysine, i.e., an amino acid which reduces the protein’s nutritional value [30,31,32,33,34,35,37]. The PDCAA index calculated for this protein group, which includes the protein digestibility, amounted to 58.5% and was lower compared to e.g., leguminous plants (69.58%) [38]. Despite such nutritional restrictions, cereal proteins (as any other proteins) may be potential BAP precursors [5,9,10,11,12,13,14,27,28,29,30].

Prolamins, globulins and other cereal proteins, irrespective of the cereal species, may be a potential source of peptide antioxidants (Table 1, Table 2, Table 3, Table 4 and Table 5) [27,28,29,30]. In α-, α/β-gliadins, the dominant bioactive sequence fragments included LH, HL, AH, LHQ, LWQ, YQL QYP and EL (no γ-gliadins), and in γ-gliadins, the LWS fragment was additionally found (Table 1). The bioactive fragment PYPQ was found in all gliadins. Moreover, all gliadins contained the most bioactive fragments IR, VY, LW, LWS and TY (only for *Triticum ascetivum*), except one analysed sequence belonging to ω-gliadins. In γ-gliadin molecules, bioactive fragments LY, LWS, PHQ, AQIPQQ, KP, VYV, MHI were found (Table 1). Such sequence fragments, after being released from wheat proteins, may be natural antioxidants. Some of the gliadin fragments presented above corresponded to antioxidant peptides isolated from food proteins [39,40,41,42,43,44,45,46,47,48,49]. Antioxidant dipeptides which contained a histidine molecule, irrespective of its location in the dipeptide (LH, HL, AH), were isolated from soybean proteins [39,40]. The LY peptide was isolated from soybean proteins as well [45]. Other fragments, such as IR and KP, were derived from egg white protein ovotransferrin [46]. The LW fragment corresponded to the antioxidant peptide isolated from the enzymatic hydrolysate of Mactra veneriformis [47]. VY and TY fragments were also found among the bioactive fragments found in proteins soluble in alcoholic solutions [48].

Bioactive fragments in the composition including glutamine (Q) and tyrosine (Y) (YQL and QYP) were isolated from casein (milk proteins) and fermented meat sauce, respectively [41,42]. The sequences LHQ, LWQ, LWS, PHQ, MHI, VYV corresponded to synthetic peptides or those obtained under molecular modelling conditions, yet they exhibited antioxidant activity [50,51]. The PYPQ fragment was isolated as a peptide from β-casein (fragment 170–173) of breast milk [44], and the fragment AQIPQQ was a typical peptide derived from pepsin hydrolysates of wheat gluten [49].

The range of the A parameter mean values, calculated for gliadins, amounted to 0.0286-0.0498 and was higher compared to glutenin proteins of the same cereal (Table 1). On the other hand, the calculated mean frequency of the occurrence of amino acid sequence fragments with antioxidant activity (the A parameter) for rice prolamins amounted to 0.0470 (Table 2) and was lower compared to the values obtained for α-gliadins (A = 0.00498), but was higher than the values calculated for other gliadins (Table 1 and Table 2). The sequences YQL, LY, IY, VW were the sequences found most frequently in rice prolamins. The latter fragment was not found in the analysed gliadin sequences, while the IY was only found in certain γ-gliadins (*Triticum aestivum*) (Table 1 and Table 2). The VW fragment found, similarly to the LW, in wheat proteins, corresponded to the antioxidant peptide isolated from a marine bivalve [47]. Similarly, the bioactive sequence IY, as well as LY bioactive fragments, were isolated from soybean proteins [45].

Glutenins, analysed among wheat proteins, were characterised by a different and more diverse potential antioxidant activity profile (Table 1). In glutenin profiles (HMW subunits), the unique motifs with antioxidant activity included eight- and nine-amino acid fragments: LQPGQGQQ and LQPGQGQQG, respectively. The former was isolated from sardine muscle, while the latter was isolated from wheat gluten [49,52]. In these subunits, a large number of potential antioxidant fragments with different structural motifs, i.e., GYY, EL, RYY, WY, WYY, SYY, LK, PW, WG, were also observed. Three-amino acid bioactive fragments (without GYY) [53] corresponded to synthetic antioxidant peptides [50]. The average frequency of the occurrence of bioactive fragments in HMW subunits composed of a larger number of amino acids amounted to 0.0372, and for units with molecules smaller by more than four times, the calculated A parameter amounted to 0.0471, with this value being comparable to the value of the A parameter calculated for rice prolamins (Table 1 and Table 2). The GYY fragment was an antioxidant peptide obtained from the hydrolysis of Okara protein using the Protease N enzymatic preparation. Okara is a minor by-product obtained during the production of soybean milk and tofu. The antioxidant activity of the peptide obtained from it was the same as that of carnosine (β-alanyl-L-histidine) [53]. The EL fragment was found in both α-, α/β- and ω-gliadins, and glutenins (Table 1). On the other hand, the PW fragment corresponded to the sequence of the antioxidant peptide isolated from buckwheat proteins after their hydrolysis using pepsin and pancreatin [54]. Another fragment (WG) was found in hydrolysed poultry protein (Table 1) [55].

In other glutenin subunits, i.e., LMW, the most frequently noted structural motifs with antioxidant activity included the sequences of LY, AH, IY, RHE, IQY, IR, VY, AY, PHQ, PWQ, PW, SVNVPLK, KP, HL and SHH (Table 1). On the other hand, antioxidant fragments of sequences of VW, LY, IY, YQL and VY were dominant in rice prolamin sequences, while AY, LY, IY, AH, EL, IR, LK, FC, TY, VY, HL, PW PWH and KP were dominant in glutelins (Table 2).

A variety of tests are used for determining the antioxidant activity of antioxidants [5,13,28,30,32,56,57,58,59]. Some of them are based on the measurement of ferric reducing antioxidant power (FRAP), oxygen radical absorbance capacity (ORAC), chelation of metals and inhibiting lipid oxidation [5,28,30,57,58,59]. Under *in vitro* conditions, the antioxidant activity is also determined based on the ability to capture free radicals (2,2-diphenyl-1-picrylhydrazyl (DPPH)), and the results are presented as the efficient concentration (EC_50_) [30]. EC_50_ indicates the concentration of an antioxidant which results in a decrease in the DPPH radical concentration by 50%. Recently, to assess the antioxidant potential of antioxidants, tests using cell cultures have been applied [13,30,56,57].

There are two main mechanisms through which antioxidant molecules can deactivate free radicals: haemolytic or hydrogen atom transfer (HAT) and single electron transfer (SET). The peptides containing tyrosine may act via the HAT mechanism, while the peptides containing cysteine, tryptophan and histidine act via the SET mechanism. In the HAT-type mechanisms, the proton bound to the heteroatom is separated. The HAT-based methods include, among others, the ORAC which measure a peptide molecule’s ability to neutralise the peroxyl radical (ROO^∙^) by donating a proton and the reactivity is determined by the bond dissociation energy (BDE) of the H-X group in the antioxidant compound. The peptides containing tyrosine, tryptophan and histidine donate a proton easily, which is of significance in ORAC tests. The presence of leucine or isoleucine may cause a steric effect or reduce histidine’s ability to donate a proton. In the hydroxyl free radical scavenging test that was conducted for the peptides derived from secalins, the presence of cysteine and its location in the analysed tripeptides (CQV, QCV, QVC, QCA) was significant for the activity degree being determined [30,57].

Wheat germ proteins hydrolysed with Proleather FG-F (a protease from *Bacillus subtilis*) removed 81% DPPH (1.6 mg/mL) and 75% O_2_^∙^ (0.6 mg/mL) radicals [30]. On the other hand, the EL fragment which was present both in α-, α/β- and ω-gliadins, and in glutenins (Table 1) was isolated from casein [43]. The activity determined for this peptide was IC_50_ = 63.1 μM (superoxide anion scavenging activity (SOSA)) based on the applied XTT {2,3-bis(2-methoxy-4-nitro-5-sulfophenyl)-5-[(phenylamino)carbonyl]-2*H*-tetrazolium hydroxide}) test [43]. On the other hand, the fragments WY and LK were often found in glutenin subunits (the HMW subunits) (Table 1), and corresponded to the peptides isolated from β-lactoglobulin (fragment 19–25) and from egg white protein ovotransferrin, respectively [46,60]. The activity of the WY peptide that was determined based on the ORAC was 7.67 μM Trolox (6-hydroxy-2,5,7,8-tetramethylchroman-2-carboxylic acid) equivalent/μM of peptide [60].

In other studies, in which rat adrenal gland pheochromocytoma (PC12) cells were treated with alcalase hydrolysates of wheat germ proteins (concentration of 1 mg/mL), they maintained their integrity (i.e., no cytotoxicity), and a 63.7% reduction in oxidative stress induced by H_2_O_2_ was observed [30]. What was also observed was the impact of the wheat peptide fraction (2 mg/mL) on the rat jejunal crypt cell line (IEC-6), which was treated with indomethacin (a nonsteroidal anti-inflammatory drug (NSAID)), with cell survivability higher by 120% compared to the cells not subjected to the NSAID. On the other hand, the RVF peptide obtained from wheat exhibited no cytotoxicity and protected the human neuroblastoma cells (SH-SY5Y) from H_2_O_2_-induced cell death, while causing an increase in their survivability by 37% [30].

In terms of their antioxidant activity, the profiled proteins also included other proteins that serve various biological functions in cereal grains (Table 1, Table 2, Table 3, Table 4 and Table 5). These proteins included enzyme inhibitors, enzymes, thaumatin-like proteins, oryzains, expansin-B1, lectins, oleosins, glycine-rich proteins, allergenic proteins, profilins, tubulin chains, germin-like proteins, actins, hordo- and avenoindolines, thaumatin-like pathogenesis-related proteins [61,62,63]. For these, the average numerical value of the calculated A parameters amounted to approx. 0.05 or was even higher than this value, and for groups of wheat proteins (thaumatin-like proteins), rice proteins (lectin, oleosins, glycine-rich proteins and tubulin chains), barley proteins (tubulin chains), and rye proteins (allergenic protein and other proteins), the range of the A bioparameter mean values amounted to 0.0308-0.0427 (Table 1, Table 2, Table 3, Table 4 and Table 5). Most of such proteins were analysed in the rice protein groups which were characterised by a very rich and diverse potential antioxidant activity profile (Table 2). Similar profiles were, however, obtained in similar groups of analysed cereal proteins, e.g., hordo- and avenoindolines were characterised by frequently occurring sequence fragments of GYY, EL, YYW, RWW, KVI, KD, RW, TW, YYL (Table 3 and Table 4). Three-amino acid bioactive fragments YYW, RWW and YYL corresponded to synthetic antioxidant peptides [50]. The bioactive fragment KVI was isolated from proteolytic digest of dried bonito [64]. On the other hand, the TW fragment corresponded to the antioxidant peptide isolated from a marine bivalve, and the RW was isolated from egg white protein ovotransferrin [46,47].

In the potential antioxidant activity profile of barley prolamins (hordeins), fragments of sequences PYPQ and PHQ as well as fragments containing up to 10 amino acid residues QKPFPQQPPF, PQIPEQF, LRTLPM, SVNVPL, LQPGQGQQ and LQPGQGQQG were found (Table 3). Apart from the last four sequences, these sequence fragments were not found in other profiled proteins (Table 1, Table 2, Table 3, Table 4 and Table 5). Biologically active fragments with the sequences of QKPFPQQPPF, PQIPEQF, LRTLPM and SVNVPL were isolated from enzymatic barley glutelin hydrolysates [65]. On the other hand, the structural motifs of LQPGQGQQ and LQPGQGQQG were found not only in hordeins but also in profiled wheat and rye glutenins (HMW subunits) (Table 1, Table 3 and Table 5). The LQPGQGQQ fragment with antioxidant activity was isolated from sardine muscle, and the second one, i.e., LQPGQGQQG was isolated from wheat gluten [49,52]. Not only was the bioactive fragment PYPQ found in hordeins but it was also present in all profiled gliadins. Such a fragment was isolated as a peptide from β-casein (fragment 170–173) of breast milk [44]. The SVNVPL fragment was present in the profiled wheat glutenins (LMW subunits) and hordeins (Table 1 and Table 3). On the other hand, the PHQ was found in hordeins, was dominant in γ-gliadins, and a smaller number of these sequences were found in alpha- and alpha/beta gliadins, and it was a synthetic peptide which exhibited antioxidant activity (Table 1 and Table 3) [50].

In oat grains, albumins, prolamins and globulins account for approx. 1–12%, 15% and 80% of total proteins, respectively. Prolamins with glutelins are storage proteins found in grain endosperm, and account for 60–70% of total proteins [31,33]. Oat prolamins (avenins) are a group of proteins with a lower lysine content compared to albumin proteins and globulins [31,32,33]. Oat proteins, due to their lower prolamin content, have a biological value higher than that of other cereals which are richer in prolamins, and the calculated Limiting Amino Acid Score (LAA) amounted to 49.8 for wheat flour and 66.9 for oat flour [32,38]. In the potential antioxidant activity profile of avenins, the VY and VYV fragments were found most frequently (Table 4). On the other hand, in the secaline profile (a single analysed sequence), only one sequence fragment with antioxidant activity (EL) was found (Table 5).

Certain peptides obtained as a result of rice endosperm protein hydrolysis, including those with the sequence FRDEHKK, significantly inhibited linoleic acid oxidation. On the other hand, the DHHQ, DAHK and DHHK tetrapeptides isolated from rice albumin exhibited antioxidant activity by inhibiting the copper ion-induced oxidation of low-density lipoprotein (LDL). Rice peptides FRDEHKK and DHHQ reduced the linoleic acid peroxidation and the Cu^2+^ copper ion-induced LDL oxidation based on chelating mechanisms. Both peptides contained histidine and aspartic acid whose presence in peptide molecules was crucial for the activity of these compounds. The FNDRLRQGQLL, GLVYIL, YHNAPGLVYIL and DVNNNANQLEPR peptides protected HepG2 cells from peroxyl radical-induced oxidative stress [30]. Not only fragments of amino acid sequences that exhibit antioxidant activity, but also other compounds found in oat grains can be nutraceuticals [32,33,56]. Oat contains from 2.3 to 8.5/100 g β-glucans, i.e., the soluble fraction of dietary fibre, that has health-promoting properties for the human body [33]. Moreover, antioxidant nutraceuticals found in oat also include tocopherols, tocotrienols, phytic acid, flavonoids and non-flavonoid phenolic compounds such as avenanthramides (AVAs) [32,33].

In the potential antioxidant activity profiles of wheat, rice, barley, oat, rye and other proteins, the most frequently found sequence fragments were composed of two or three amino acids (Table 1, Table 2, Table 3, Table 4 and Table 5). Certain fragments of the sequence were fragments with antioxidant activity only, and such sequence fragments included e.g., EL, LWQ, LHQ, PHQ, YQL, QYP, RYY, WYY, SYY and PYPQ as well as fragments containing up to 10 amino acid residues QKPFPQQPPF, PQIPEQF, LRTLPM, SVNVPL, LQPGQGQQ and LQPGQGQQG. The fragments composed of two amino acid molecules were fragments that may exhibit more than one biological activity, e.g., they may be inhibitors of various enzymes including angiotensin-converting enzyme (EC 3.4.15.1) (ACE inhibitor), calmodulin-dependent phosphodiestesterase 1 (EC 3.1.4.17) (CaMPDE inhibitor), dipeptidyl peptidase IV (DPPIV inhibitor) (EC 3.4.14.5), renin (EC 3.4.23.15) [20,28,29,61].

### 3.2. Bioevaluation of the Plant Protein Proteolysis Simulation Process and Products

To carry out the tested plant protein proteolysis simulation, ficin (EC 3.4.22.3), stem bromelain (EC 3.4.22.32) or pepsin (pH > 2) (EC 3.4.23.1) were used. All of these enzymes, due to their biological activity, are also classified as nutraceuticals [2,3,7] and used not only in medicine but also in the food industry in which they are utilised *inter alia* to tenderise meat [8,25,26]. Pepsin is also an enzyme synthesised in the human gastrointestinal tract [8]. However, the broadest biological action spectrum is exhibited by bromelain found in the pineapple, whose content is the highest in the stem from which it is isolated more often. The enzyme exhibits anti-oedematous and anti-inflammatory activity, inhibits platelet aggregation, prevents vein thrombophlebitis, results in an increased absorption of drugs, particularly antibiotics, can break up cholesterol plates, and alleviates degenerative joint disease symptoms. It is used in the treatment of cerebrovascular diseases, CVD diseases, acute inflammation and sports injuries. Bromelain exhibits an analgesic effect by affecting pain mediators (bradykinin). Moreover, animal studies confirmed that it maintains its proteolytic activity in the gastrointestinal tract despite the presence of acid-neutralising drugs, e.g., sodium bicarbonate. The enzyme is active within the pH range of 5.5–8. Approx. 40% of the enzyme in the high-molecular form is absorbed in the gastrointestinal tract. This enzyme’s activity was also detected in blood plasma [2,6].

Ficin (EC 3.4.22.3) which was used to carry out the proteolysis simulation recognised and hydrolysed the C-terminal peptide bonds in the polypeptide chain that contained the amino acids K-, F-, Y-, G-, S-, L-, R- and H-. On the other hand, stem bromelain (EC 3.4.22.32) recognised and hydrolysed bonds in which V-, A-, T-, L-, R-, G-, S-, F- were found, from the same side of the peptide bond as ficin. Pepsin (pH > 2) (EC 3.4.23.1) hydrolysed the C-terminal peptide bonds with the participation of such amino acids as F-, L-, G-, Y-, A-, E-, Q-, T-, N-, K-, D- and M-, and the N-terminal peptide bonds with the participation of -V and -I [61].

The dominant protein groups found in cereal grains are prolamins and globulins, of which wheat, barley and rye prolamins account for 30-50% of all proteins [30]. In oat and rice grains, such groups correspond to approx. 4-15% of total proteins [30,31,32], which implies that the number of bioactive peptides potentially released from these protein groups may be the highest compared to other analysed protein groups.

Among ficin hydrolysates, i.e., γ-gliadins (*Triticum aestivum spp. sphaeroccoum, Triticum aestivum spp. compactum*) and thaumatin-like proteins, no peptide antioxidant was present, and pepsin did not release any peptide with the desired activity from this second protein groups either (Table 6). Bromelain released no antioxidant peptide from barley thaumatin-like proteins, while pepsin (pH > 2) released no antioxidant peptide from hordoindolines and buckwheat legumin and vicilin proteins (Table 8 and Table 10). No peptide antioxidants were released from certain oat proteins (thaumatin-like pathogenesis-related proteins) whose simulation used ficin or bromelain (Table 9). As a result of the monocatalytic proteolysis simulation carried out, none of the three enzymes used released an antioxidant peptide from secalins (Table 10).

Ficin hydrolysed gliadins to a lesser extent (DH_t_ 24–28%) than glutenins (HMW and LMW subunits) DH_t_ 29–39%. Although ficin broke down more peptide bonds in rice prolamins DH_t_, i.e., approx. 41%, and 43% in rice glutelins, more antioxidant peptides were released from wheat proteins, which may also result from the fact that more proteins were subjected to the proteolysis simulation (Table 6 and Table 7).

Although in ficin hydrolysates of gliadins and glutenins (HMW and LMW subunits), peptides with sequences of AH, EL, AY and IR were found, peptide antioxidants TY and PEL were additionally found in gliadins, while WG, PWS and WY were found in glutenins. The obtained antioxidant peptide profile resulted from the specificity of action of the enzyme used for hydrolysis and amino acid formation of the protein monomers being hydrolysed (Table 6). Ficin hydrolysed approx. 35% of the bonds found in hordeins, and more than 43% in hordothionins and other proteins. The hydrolysis products included the EL, PWS, AY and WY biopeptides and a unique PQIPEQF fragment which was isolated under *in vitro* conditions from barley protein hydrolysates [65].

Of all the enzymes and main cereal proteins used to carry out the proteolysis simulation, most antioxidant nutraceuticals were obtained as a result of the use of pepsin (pH > 2) from gliadins and glutenins (HMW and LMW subunits) (Table 6, Table 7, Table 8, Table 9 and Table 10). Among the released biopeptides, the peptides with sequences of HL, VY, PHQ, IY, PWQ, WG and WY were dominant (Table 6). Many nutraceuticals were also released by bromelain from gliadins and glutenins (HMW and LMW subunits), and the potential antioxidant nutraceutical sequences that were noted the most repeatedly included YQL, EL, IR, HL, WG, YYL, YYS, PWS. In the obtained peptide nutraceuticals, H, Y and W amino acid residues were present. The presence of the Y, W, C or M residues in the antioxidant dipeptide structures played a crucial role in capturing free radicals, thus considerably affecting the antioxidant activity of these compounds [27,28,50,60]. The study which used the quantitative structure–activity relationship (QSAR) model for the molecular modelling confirmed that the presence of W, Y or C in the N-terminal location is of significant importance to the antioxidant activity of peptides [5,59]. W or Y amino acids were found in antioxidant nutraceuticals (WG, YYL, YYS, YQL) which were released from gliadins and glutenins (HMW and LMW subunits) during the hydrolysis simulated by bromelain. Based on the calculated A_E_ parameter mean values, it can be concluded that nutraceuticals were more frequently released from rice prolamin protein structures (A_E_ = 0.0118), and less frequently released from barley (0.0074) and other cereal protein species, which may be of significance in the context of designing nutraceutical food (Table 7, Table 8, Table 9 and Table 10). On the other hand, antioxidant peptide nutraceuticals IY and VY were most frequently released from rice prolamins and glutelins by pepsin (pH > 2.0) and ficin (Table 7). Various scientific studies have confirmed that the chemical structure of peptides has a significant importance for the activity of peptide antioxidants [5,27,28,30,50,57,59,60].

## 4. Materials and Methods

### 4.1. Materials

The study used 416 plant protein monomers or/and their fragments, whose amino acid sequences were recorded using a single-letter amino acid code were derived from the base “Proteins” found in the BIOPEP-UWM database resources (http://www.uwm.edu.pl/biochemia/ index.php/pl/biopep) [61]. Based on the biologically active amino acid sequences defined as biologically active peptides and deposited in the base “Bioactive peptides” which are an integral part of the BIOPEP-UWM database resources, bioactive fragments of sequences with potential antioxidant activities were searched for in the analysed proteins. The BIOPEP-UWM database program compared the analysed protein sequence with 629 amino acid bioactive fragments with antioxidant activity deposited in the base “Bioactive peptides” which currently (data as at 10 December 2019) contains 3,820 biopeptides with 43 different activities. For the bioinformatic analysis, the following proteins were selected: wheat (*Triticum aestivum, Triticum aestivum subsp. Spelta, Triticum aestivum ssp. sphaerococcum, Triticum aestivum ssp. compactum, Triticum aestivum spp. tibeticum, Triticum turgidum subsp. durum*) (190), rice (*Oryza sativa, Oryza sativa* [japonica cultivar-group], *Oryza sativa subsp. japonica*) (112), barley (*Hordeum vulgare*) (59), oat (*Avena sativa*) (23), buckwheat (*Fagopyrum esculentum, Fagopyrum gracilipes, Fagopyrum tataricum*) (13), rye (*Secale cereale*) (16), sorghum (*Sorghum vulgare*) (3), and 629 antioxidant peptides were used [61].

### 4.2. Methods

Selected plant proteins were characterised as sources of antioxidant bioactive fragments based on the obtained profiles of potential antioxidant activity of proteins, including the calculation of the frequency of occurrence of a bioactive fragment with a given activity in the protein sequence (the A parameter). A proteolysis simulation (*in silico* proteolysis, *in silico* protein hydrolysis) was carried out with the use of selected enzymes, and a bioevaluation of proteolysis products was conducted using unique and innovative bioinformatic tools available in the BIOPEP-UWM database [20].

#### 4.2.1. Characteristics of Plant Proteins as Precursors of Bioactive Fragments with Antioxidant Activity

##### Profiles of Potential Antioxidant Activity of Selected Plant Proteins

For each analysed protein or protein fragment, a profile of potential antioxidant activity was generated. To this end, after entering the base “Protein” which is part of the BIOPEP-UWM database resources, the “Analysis” tab was selected, followed by the “Profiles of potential biological activity”, and then the activity “antioxidative” was selected from the window “Select activity” and after unfolding the list of activities, and the analysed protein (protein sequence) from the base “Protein database” (after unfolding the list of proteins). In this way, the data search was limited exclusively to antioxidant activity. The profile of potential biological activity is defined as the type and location of bioactive fragment in a protein chain, and, if no specific type of activity is selected at the stage of generating a profile of potential biological activity of protein, then all possible profiles of potential various activities of protein will be generated, and since the base “Bioactive peptides” currently contains 3,820 sequences of biologically active peptides (bioactive peptides, biopeptides) with 43 different bioactivities (data as at 10 December 2019), a large volume of various biological data, not necessarily of significance to the established bioinformatic experiment, could be obtained in this way (http://www.uwm.edu.pl/biochemia/index.php/pl/biopep) [61].

##### Calculation of the Bioparameters Characterising the Antioxidant Activity of Selected Plant Proteins

Based on the profile of potential antioxidant activity generated for each analysed protein, protein can be characterised in terms of the bioactivity under study, but it is difficult to assess protein as a source of antioxidant peptides and compare it to other analysed proteins, therefore, in order to carry out a quantitative bioevaluation of particular proteins, the A parameter (which takes into account the number of amino acid residues in the protein chain) was calculated. The A parameter is defined as the frequency of bioactive (antioxidative) fragments occurrence in a protein sequence [20,66]:

A = a/N(1)

where a—the number of fragments with a given activity (antioxidant), N—the number of amino acid residues.

To calculate the above-mentioned parameter, similarly to the determination of the profiles of potential antioxidant activity, the tools available in the BIOPEP-UWM database were applied. To this end, the base “Proteins” was selected, followed by the tab “Analysis” and “Calculations”. The activity “antioxidative” was then selected from the window “Select activity” after unfolding the list of activities, and the analysed protein (protein sentence) was selected from the base “Protein” (after unfolding the list of proteins) [20,61].

#### 4.2.2. *In Silico* Hydrolysis of Selected Plant Proteins with the use of Selected Enzymes (BIOPEP-UWM)

A simulation of proteolysis of the analysed proteins was conducted using two plant enzymes and one animal enzyme out of 33 different proteolytic enzymes with various specific effects on the protein molecule available in the BIOPEP-UWM database. The *in silico* hydrolysis was carried out with ficin (EC 3.4.22.3) or stem bromelain (EC 3.4.22.32) or pepsin (pH > 2) (EC 3.4.23.1), i.e., enzymes that had been assigned identification numbers (IDs) of 25, 30 and 39, respectively, in the BIOPEP-UWM database [61].

A total of 1248 proteolysis simulations were conducted using the application “Enzyme(s) action”. The application is available after opening the panel “Analysis” which was made available to BIOPEP-UWM database users only after the previous entering one of the bases making up its resources, e.g., “Proteins” or “Bioactive peptides”. The tab “Protein database” was then opened, and the analysed molecule and enzymes were selected from the list of proteins and enzymes, respectively. Database users could select an enzyme only after unfolding the list of enzymes provided in the window “Select enzymes”. Then, by selecting “View the report with the results”, “BIOPEP: Report of enzyme action” was obtained [20,61].

##### Characteristics of Antioxidant Peptides Obtained from the Performed Proteolysis Simulation

To obtain data on the released antioxidant peptides, the “Search for active fragments” tab was used. After opening the tab, a report was generated. The report contained data on biopeptides (with different activities) obtained from the performed monocatalytic proteolysis simulation and concerning *inter alia* the following: own identification number (ID) of biopeptide (deposited in the “Bioactive peptides” database), sequence, location in a protein sequence, name, function and activity. The conducted bioinformatic study selected only the peptides with antioxidant activity based on the amino acid sequence, ID, activity and the location of biopeptide in the protein polypeptide chain [20,61].

##### Bioevaluation of the Proteolysis Simulation Process and Products

**Figure 2:**
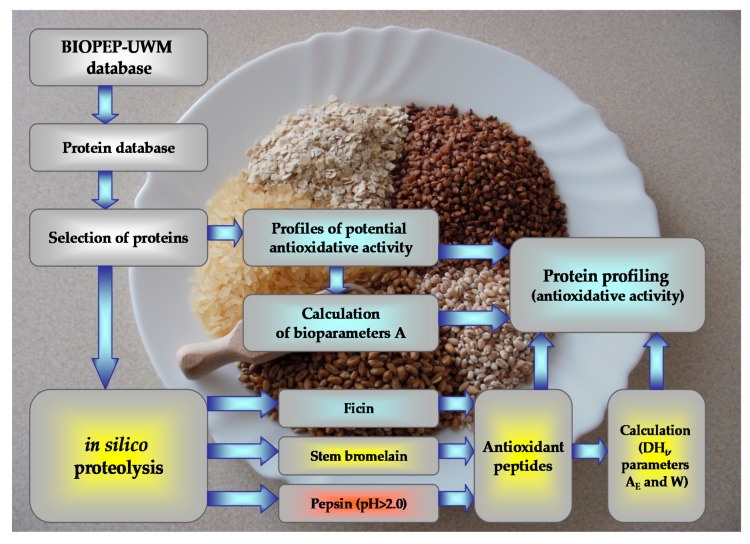
The procedure scheme.

To carry out a bioevaluation of the process and products obtained from the simulated enzymatic degradation of plant proteins, the theoretical degree of hydrolysis (DH_t_) [20,61,67] was calculated as well as the quantitative parameters that characterise proteolysis, including the frequency of the release of fragments with a given activity by selected enzymes (A_E_ parameter) and the relative frequency of release of fragments with a given activity by selected enzymes (W parameter) (Figure 2) [20,61,68].

The theoretical degree of hydrolysis (DH_t_)

DH_t_ = d/D × 100%, (2)

where d—number of hydrolysed peptide bonds in a protein/peptide chain, D—total number of peptide bonds in a protein/peptide chain

The frequency of release of fragments with a given activity (antioxidant) by selected enzymes (A_E_ parameter)

A_E_ = d/N (3)

where d—the number of peptides with a given activity (antioxidant) released by a given enzyme (e.g., ficin), and N—the number of amino acid residues in protein

The relative frequency of release of fragments with a given activity (antioxidant) by selected enzymes (W parameter)

W = A_E_/A (4)

A_E_—the frequency of release of fragments with a given activity (antioxidant) by selected enzymes (from Equation-3), A—the frequency of bioactive fragments occurring in a protein sequence (from Equation (1)).

## 5. Conclusions

Cereal grains are part of the staple diets of humans. The proteins found in cereal grains (a content of up to 20%) are incomplete proteins (a low exogenous amino acid content) compared to animal proteins. However, given the high availability and lower costs of acquiring such plant raw material in many regions worldwide, they are an attractive protein source from which peptide nutraceuticals that eliminate reactive oxygen species can be released. This paper presents new/other possibilities for the use of cereal proteins, including allergenic and enzymatic proteins as well as inhibitors of certain cereal enzymes that can be used to produce antioxidant nutraceuticals. To obtain such bioactive compounds, enzymes which are also nutraceuticals, i.e., ficin, bromelain and pepsin (pH > 2.0), were used. In nature, these three enzymes are found in fig trees (ficin), pineapples (bromelain) and digestive tracts (pepsin (pH > 2)). Moreover, they are used in the food industry and are also ingredients of dietary supplements, *inter alia* those improving protein digestion in the human digestive tract.

During the study, to release nutraceuticals from protein structures, resources of the BIOPEP-UWM database (amino acid sequences of cereal proteins–the “Proteins” database, and antioxidant peptides–the “Bioactive peptides” database), as well as selected tools available in it, were used to perform a simulated proteolysis. The data contained in the BIOPEP-UWM database are updated on an ongoing basis, and new bioinformatic tools are introduced to its resources. Moreover, new sequences of biopeptides, including antioxidant peptides, are continuously deposited in the database. This highlights the innovation of *in silico* testing in the analysis of biopeptides with various effects on the human body.

The study, whose results are presented in this paper, reflects the current state of knowledge about cereal proteins as peptide nutraceutical precursors, obtained from proteins of various cereal species, and the possibilities for obtaining such bioactive compounds under conditions of hydrolysis simulated by selected enzymes and those classified as compounds referred to as nutraceuticals. During the testing, cereal proteins were subjected to “specific scanning” that was carried out in the BIOPEP-UWM database, to find the so-called permutations with repetitions which indicate the number of fragments and the sequence with antioxidant activity (based on amino acid sequences of antioxidant peptides deposited in the “Bioactive peptides” database, which are the BIOPEP-UWM database resources). Based on the calculated frequency of the occurrence of sequence fragments with antioxidant activity (the A parameter), monomer molecules from cereal proteins of various species were compared. The A parameter numerical value, calculated for a single protein monomer molecule, includes both the number of sequence fragments with antioxidant activity and the number of amino acid molecules found in the protein molecule under analysis. Based on the numerical values of the A parameters, the analysed proteins can be compared while indicating which of the analysed proteins or protein groups can be a potential, better precursor or source of antioxidant peptides (nutraceuticals).

Of all the analysed cereal protein monomers (all cereal species under analysis), the best source of antioxidant peptide nutraceuticals was barley hordoindoline monomers as well as a protein group of “actins and other rice proteins”. On the other hand, of various analysed groups and species of cereal protein monomers, while taking into account the so-called “main proteins” found in grains, the best protein monomers from which antioxidant peptides can be released included kafirins (sorghum proteins), rice glutelins and alpha-gliadins (wheat proteins), i.e., proteins with a very diverse amino acid sequence profile.

Based on the averaged A parameter values calculated for monomers of all the analysed protein groups for the cereal species under study, the best precursors of peptide nutraceuticals included proteins in the following order: sorghum (three sequences were analysed) > barley > rice > oat > wheat > buckwheat > rye.

In the potential antioxidant activity profiles of wheat, rice, barley, oat, rye and sorghum, the most frequently found sequence biological fragments were those composed of two or three amino acids. Some of them were fragments with antioxidant activity only, e.g., EL, LWQ, LHQ, PHQ, YQL, QYP, RYY, WYY, SYY and PYPQ, similarly to sequence fragments containing up to 10 amino acid residues QKPFPQQPPF, PQIPEQF, LRTLPM, SVNVPL, LQPGQGQQ and LQPGQGQQG. Sequence fragments composed of two amino acid molecules were multi-active fragments which may exhibit more than one biological activity, e.g., they can be enzyme inhibitors (ACE, CaMPDE, DPPIV, renin) (the sequences of obtained antioxidant nutraceuticals were compared to sequences of other biopeptides available in the “Bioactive peptides” database).

In hordein profiles, biological fragments that are very characteristic of this protein group, namely PHQ, PYPQ, QKPFPQQPPF, PQIPEQF, LRTLPM, SVNVPL, LQPGQGQQ and LQPGQGQQG, were found. The two latter bioactive fragments were also found in wheat and rye glutenins (HMW subunits). On the other hand, PYPQ and SVNVPL were found in all properly profiled gliadin and glutenin groups (LMW subunits).

Of the three enzymes and main cereal proteins used to carry out the proteolysis simulation, most antioxidant nutraceutical molecules were obtained as a result of the use of pepsin (pH > 2) from gliadins and glutenins (HMW and LMW subunits), but it is the number of analysed wheat protein sequences that was the highest as compared to other proteins under study. Among the released biopeptides, the peptides with sequences of HL, VY, PHQ, IY, PWQ, WG and WY were dominant. Thirteen WG molecules were released from glutenins (HMW subunits) by both pepsin (pH > 2.0) as well as ficin and bromelain. Many nutraceutical molecules were also released by bromelain from gliadins and glutenins (HMW and LMW subunits), and the potential antioxidant peptide nutraceutical sequences that were noted the most repeatedly, and in which tyrosine and histidine molecules were frequently found attached to the N-terminus, included YQL, EL, IR, HL, WG, YYL, YYS and PWS. The antioxidant EL nutraceuticals were released by ficin and bromelain from wheat proteins but the former enzyme released more such antioxidant peptide molecules. On the other hand, both enzymes released PWS nutraceuticals from the same proteins, and most IR biopeptides were released from gamma-gliadins and glutenins (LMW subunits) were released by bromelain.

Of all the analysed prolamins of various cereal species, pepsin (pH > 2) released the most nutraceuticals from rice protein monomers, and fewer of them from barley, wheat, sorghum (kafirins) and oat (avenins). IY and VY were the antioxidant peptide nutraceuticals most frequently released from rice prolamins and glutelins by pepsin (pH > 2.0) and ficin (the same number of nutraceutical molecules was released by both enzymes), and YYG was released from rice prolamins by bromelain. On the other hand, PWQ, VY, PHQ and RHE were biologically active peptides released as a result of simulated hordein hydrolysis by pepsin (pH > 2), and the unique, seven-amino acid nutraceutical PQIPEQF was released by ficin.

Both the hydrolysis carried out with enzymes regarded as nutraceuticals and the products resulting from their action, including antioxidant peptide nutraceuticals, are considered to be safe processes and products. Currently, computer testing plays an important role in food and nutrition sciences despite certain limitations resulting from their use. The results of *in silico* testing must be confirmed by laboratory tests, and the amino acid sequences of peptide nutraceuticals by tests using mass spectrometry techniques. However, the results of this study can be used to design innovative food ingredients including antioxidant nutraceuticals and a new generation of food intended to satisfy the specific needs of the body. What is more, they can be used to design diets as well as various technological processes in which the presence of antioxidant peptide nutraceuticals is advisable. It is also possible that certain antioxidant peptides may also exhibit a synergistic effect towards either other biopeptides or other biologically active compounds found in food, thus enhancing their biological impact. This aspect of scientific considerations requires further research to be carried out using a variety of experimental techniques.

## Figures and Tables

**Table 1:** Characteristics of wheat proteins antioxidant activity (^I^Triticum aestivum, ^II^Triticum aestivum subsp. Spelta, ^III^Triticum aestivum spp. sphaeroccoum, ^IV^Triticum aestivum spp. compactum, ^V^Triticum aestivum spp. tibeticum, ^VI^Triticum turgidum subsp. durum).

Protein Groups (n^T^)	NAAR* x (n^c, f^) (Range)	NAFS** x (Range)	A*** x (Range)	AAS**** (Antioxidant Activity)
^I,II^Alpha-gliadins (21^T^, 20^I^ + 1^II^)	281 (16^c^ + 2^f^) (259–318)	14 (10–19)	0.0498 (0.0314–0.0696)	L**H** (1)^16^, L**HH** (1)^2^, **H**L (1)^12^, A**H** (1)^13^ (2), **HH** (2)^2^ (3), EL (1)^12^, PYPQ (1) (2)^11^, (3)^5^ (5), LHQ (1)^14^, LWQ (1)^14^, IR (1)^18^, TY (1)^16^, VY (1)^18^, LW (1)^14^, LLR (1)^2^, YQL (1)^16^, QYP (1)^16^ (2)^2^, KYL, RW, AY, LY, **HHH** (1)^2^ (2), P**H**Q (1)^2^, Q**HH**
^I^Alpha/beta-gliadins (12^T^)*****	287 (10^c^ + 1^f^) (186–319)	13 (10–19)	0.0463 (0.0313–0.0653)	L**H** (1)^8^, L**HH**, **H**L (1)^3^, A**H** (1)^8^ (2), **HH** (2) (3), EL (1)^3^, PYPQ (2) ^2^ (3) ^7^ (5), L**H**Q (1)^7^, LWQ (1)^7^, IR (1)^11^, TY (1)^10^, VY (1)^10^, LW (1)^7^, YQL (1)^9^, QYP (1)^9^ (2)^2^, Q**HH**, **HHH** (1), (2), AY, PHQ, FC, KYL, MY
^I^Gamma-gliadins (32^T^)******	276 (21^c^ + 8^f^) (203–337)	10 (2–13)	0.0349 (0.0078–0.0476)	**H**L, AY (1)^2^, LY (1)^11^, IY (1)^2^, A**H** (1)^4^, P**H**Q (1)^7^ (2)^19^, PWQ, PW (1)^4^, IR (1)^21^ (2)^6^, SVNVPL (1)^2^, LWS (1)^20^, AQIPQQ (1)^23^, KP (1)^20^, VY (1)^23^, LW (1)^21^, VYV (1)^23^, LWQ, RYQ, M**H**I (1)^11^, TY (1)^5^, VPW (1)^3^, PYPQ (1)^12^, **HH**, F**HH**, LK (1)^3^, FC, YLL (1)^4^
^III,IV^Gamma-gliadins (6^T^, 4^III^+2^IV^)	265 (6^f^) (244–283)	12	0.0455 (0.0424–0.0492)	LY (1)^6^, PYPQ (1)^6^, LWS (1)^6^, P**H**Q (2)^6^, AQIPQQ (1)^6^, IR (1)^6^, KP (1)^6^, VY (1)^6^, LW (1)^6^, VYV (1)^6^, M**H**I (1)^6^
^I^Omega-gliadins (3^T^)	280 (1^c^)	8	0.0286	EL (3), PYPQ (2), PWQ, PEL, PW
^I^Glutenin, HMW subunits (12^T^)	734 (12^c^) (462–973)	28 (16–43)	0.0372 (0.0242–0.0477)	LQPGQGQQ (2)^5^, LQPGQGQQG (2)^5^, GYY (9)^5^ (10) (12) (17)^2^ (18) (20) (22), EL (1)^5^ (2)^3^ (3)^4^, WY (1)^7^ (2), WYY (1)^7^ (2), RYY (1)^6^, YYL (1) (2)^5^, YYS (1)^5^, SYY (1)^11^, PWY (1)^4^, PW (1)^10^, LK (2)^2^ (3)^4^, WG (1)^12^, ACQ (1)^4^, PWS (1)^6^, MM, L**H**, **H**L, PYY, L**H**Q, R**H**Y, QYP (4), LWG (1)^2^, P**H**Q (1)^2^, KP (1)^2^ (2), LW (1)^2^, IY, YYI, YYQ, P**H**Y, R**H**V, IR, EYY
^I^Glutenin, HMW subunits (21^T^)	178 (1^c^ + 19^f^) (101–238)	9 (3–16)	0.0471 (0.0142–0.0672)	LQPGQGQQ, LQPGQGQQG, GYY (1)^4^ (2)^8^ (3)^3^ (6) (7), LK (1)^5^ (2)^4^ (3), EL (1)^7^ (2)^3^ (3)^5^, RYY (1)^9^ (2)^2^, SYY (1)^10^, WG (1)^13^, ACQ (1)^7^, PWS (1)^4^, PW (1)^5^, AY (1)^2^, LLR (1)^6^, YYL (1)^2^ (2)^2^, LWQ, EYY (1)^2^, LWG (1)^2^, PWQ, LW (1)^3^, WY (1)^2^, WYY (1)^2^, YYS, AW, PYY
^I,V^Glutenin, LMW subunits (57^T^, 54^I^+2^V^)	319 (43^c^ + 13^f^) (212–392)	9 (3–17)	0.0278 (0.0131–0.0466)	LY (1)^51^, AH (1)^18^, IY (1)^24^, R**H**E (1)^12^, IQY (1)^12^, IR (1)^55^, VY (1)^29^ (2)^6^, AY (1)^27^, PHQ (1)^41^ (2)^9^, PWQ (1)^23^, PW (1)^23^, SVNVPL (1)^32^, TY (1)^8^, LRTLPM (1)^3^, MM (1)^5^, LPM (1)^5^, PHK, WY (1)^4^, LWQ (1)^4^, LWY (1)^4^, KP (1)^10^, LW (2)^4^, **H**L (1)^15^, L**H** (1)^3^, **HH** (1)^8^ (2)^4^, GPP (1)^3^, EL (1)^2^ (2), S**HH** (1)^10^, **HHH** (1)^4^, L**H**N (1)^2^, P**H**L, R**H**Q, LHQ, T**HH**
^I^Enzyme inhibitors (10^T^)	135 (9^c^) (121–168)	7 (4–11)	0.0548 (0.0280–0.0887)	LY (1)^5^, EL (1)^4^ (2)^3^, YCY (1)^2^, KD (1)^5^, LK (1)^3^, HL, LLPH, IY, YVL, KP, LW, LWI, PW (1)^4^, YVE, A**H**, MY (1)^2^, VKL (1)^3^, VVKL (1)^2^, AY (1) (2)^2^, LLR, CGA (1)^2^, PWS, VY, GYY (1)^2^, YYL (1)^2^, PEL (1)^2^, TY (1)^2^, EQC (1)^2^
^I^Thaumatin-like proteins (3^T^)	167 (3^c^) (154–173)	5 (2–8)	0.0308 (0.0116–0.0519)	AY (1)^2^, FC (1)^3^, LK, TW, VW (3), WG, **HH**P, Y**HH**, **HH**, P**H**G
^I^Other (13^T^, 12^I^+1^VI^)	173 (6^c^ + 2^f^) (118–253)	9 (4–16)	0.0474 (0.0294–0.0632)	AY (1) (2), LY (1)^3^, FC (1)^4^ (2)^2^, YVE (1)^3^, EQC, EL (1)^2^, VKL, VKV, LK (1)^3^ (3), KP (1)^5^, TY, VW (1)^2^, KD (1)^5^, YGY (1)^2^ (2), WG (2)^3^, CGA (2)^3^, GAA (1)^2^, MM (1)^2^, IY, PWV, KAI, KVI, VPW, PW, LKP
*****ID 1365 (^f^120 NAAR, 13 NAFS, A = 0.1083, L**H**, **HH** (3), Q**HH**, **HHH** (2), L**H**Q, LWQ, IR, VY, LW, QYP); ******ID 1364 (^f^146 NAAR, 14 NAFS, A = 0.0959, L**H**, **H**L (2), AY, LY, A**H**, **E**L, L**H**Q, LWQ, P**H**Q, TY, LW, YQL, SVNVPL)				

n^T^—the number of total proteins including all protein fragments available from the BIOPEP-UWM database (access on August/September 2019 and from the 1st to the 10th December 2019. The data were updated as of 10 December 2019), n—the number of analysed proteins including selected fragments, ^c, f^—protein sequence status (^c^-complete, ^f^-a protein fragment), x—a mean value, *NAAR—the number of amino acid residues found in the analysed protein or protein fragment, **NAFS—the number of potential sequence fragments with an antioxidant activity, ***A—the A parameter (the frequency of bioactive fragment occurrence in a protein sequence) (defined in the “Materials and Methods” section of the study), ****AAS—amino acid sequence presented in the form of a single-letter amino acid code: A—Alanine, R—Arginine, N—Asparagine, D—Aspartic acid, C—Cysteine, Q—Glutamine, E—Glutamic acid, G—Glycine, H—Histidine, I—Isoleucine, L—Leucine, K—Lysine, M—Methionine, F—Phenylalanine, P—Proline, S—Serine, T—Threonine, W—Tryptophan, Y—Tyrosine, V—Valine. The number of bioactive fragments with a particular amino acid sequence found in a single analysed protein is provided in brackets. The number of proteins in which the particular bioactive fragment(s) was (were) found is provided in the superscript. The histidine residues found in the bioactive sequences are highlighted in bold, *****, ******—the results provided in Table 1 do not include the data originating from analyses of two proteins (IDs 1365, 1364—classified into different groups of wheat proteins), even though they satisfied the conditions of the adopted molecular criterion. Results for both proteins are presented below Table 1. HMW—high molecular weight, LMW—low molecular weight.

**Table 2:** Characteristics of rice proteins antioxidant activity (^I^*Oryza sativa,* ^II^*Oryza sativa* [japonica cultivar-group], ^III^*Oryza sativa subsp. japonica*).

Protein Groups (n^T^)	NAAR* x (n^c, f^) (Range)	NAFS** x (Range)	A*** x (Range)	AAS**** (Antioxidant Activity)
^I^Prolamins + globulins***** (11^T^)	144 (9^c^ + 2^f*****^) (101–174)	7 (3–11)	0.0470 (0.0297–0.0809)	AY (1)^3^, EL (1) (1)***** (4)*****, VW (1)^5^, LY (1)***** (1)^3^, RYY (1)^3^, RYQ, IY (1)^6^, YYG (1)^3^, YQL (1)^7^, WY (1)^3^, VY (1)***** (1) (2)^2^, LQGM (1)^2^, MM (1)***** (4)^2^, **H**L (1)***** (1)^3^, YYS, NYY, AH*****, P**H**W*****, R**H**L*****, GGE (1)^2^*****, LK (1)^2^*****, R**H**Q*****, IR*****, KP*****, P**H**L
^I^Glutelins (16^T^)	500 (7^c^) (496–510)	28 (23–30)	0.0551 (0.0461–0.0602)	LLP**H** (1)^2^, AY (1)^4^ (2)^2^ (3), LY (1)^5^ (2)^2^, IY (1)^4^ (2)^2^, K**H**NRGDEF, A**H** (2)^6^, (3), EL (1)^3^ (2)^2^ (3) (4), P**H**Y (1)^2^, VKV (1)^2^, KD, IR (1)^6^ (2), LK (1) (2)^2^ (3)^3^, TY (1)^3^ (2) (3)^2^, VY (1) (2)^2^ (3) (4) (5)^2^, FC (1)^2^ (2)^4^, VYV (1)^3^, **H**L (1)^5^, **HH**, IKK, Q**HH**, YRY, R**H**R, YVY (1)^3^, QYY (1)^3^, PW**H** (1)^4^, PWN, PW (1)^4^, RW (1)^3^, LKP (1)^3^, KP (1)^4^, MY (1) (2), RYQ, LLR, FLPE, R**H**I, RWN (1)^2^, EAK, L**H** (2), A**H**K, EYY, YYN, YSY, L**H**Q (2), P**H**G, P**H**W, R**H**V, TW, AW, LPM
^I^Oryzains + ^II^expansin -B1****** (4^T^, 3^I^+1^II^)	390 (4^c^) (267–471)	21 (18–23)	0.0554 (0.0467–0.0749)	AY (1)****** (1)^2^, LY (1) (2), A**H** (1) (2), EL (1)****** (1) (2) (3), R**H**D, GGE (1)****** (1), KD (2)^2^ (4) (5)******, LK (1) (2)****** (2)^2^, KP (1)^2^ (2) (3)******, VW, LW, D**H**G (1)^2^, WG (1)****** (2) (3)^2^, MM, CLV, IY, YEY, YYT, NYY, YCY, P**H**E, TY, AW, R**H**G, MY, VKV, IR, VY (1)****** (1), N**H**AV, GAA, GPP (1)****** (1), LAC******, WY******, EAK******, TW******
^I^Lectin + oleosins (3^T^)	182 (3^c^) (148–227)	6 (5–7)	0.0312 (0.0291–0.0338)	EL (2), WG, YGY, P**H**N, FC (2), YKY, MY, LK, MM, KYL, A**H** (2), YYQ, QYY, YVE
^I^Glycine-rich proteins + other1 (9^T^)	161 (9^c^) (135–183)	7 (3–14)	0.0427 (0.0185–0.0795)	YGY (6), KVI, **H**L, **HH**, A**H**, G**HH**, GGE (2), GA**H**, GAA (1)^2^ (2), EL (1)^5^, P**H**R, KD (1)^2^, IR (1) (3), KP (1)^2^, ELLI, FKK, YYV, SYY, P**H**V, PWG, YVL, PW, VY, AW (1)^3^, WG (1)^2^, TY (1)^4^, AY, TW, VW (2), FC, L**H**, L**H**N, VKL, LTC, LPM, P**H**A, P**H**N
^I^Allergenic proteins + homolog protein + ^II^profilin (13^T^, 12^I^ + 1^II^)	145 (8^c^+4^f^) (109–166)	8 (3–18)	0.0566 (0.0275–0.1259)	**HH** (1)^8^, Y**HH**, IY (1)^11^, A**H**, EL (1)^8^ (2) (3), D**HH** (1)^6^, MY (1)^5^, KD (1)^5^, VY (1)^7^, FC (1)^11^, D**HH**Q (1)^2^, R**H**E, RWT, RW, D**HH**QVYSPGEQ, TY (1)^3^, VW (1)^2^, CGA, EQC, L**H**, **H**L (1) (2), YYN, LY (3), LYY, YLY, L**H**S, P**H**D, RWW, IRW, RW (1)^2^, IR (1)^2^, G**HH**, KP
^I^Tubulin chains (5^T^)	451 (5^c^) (444–469)	19 (14–21)	0.0413 (0.0311–0.0473)	AY, LY (1)^5^, IY (2), A**H**, L**H** (1) (3)^3^, EL (1) (2)^3^, WY (1)^4^, R**H**G, MY (2), KD (2)^2^, TY (1)^2^, MM (1) (2)^3^, **H**L (1)^3^ (2), YYN (1)^3^, VYY (1)^3^, L**H**F (1)^3^, L**H**I (1)^3^, L**H**W (1)^3^, R**H**G (1)^3^, PEL (1)^3^, IR (1)^3^ (3), LK (1)^2^, (2)^2^, VY (1)^3^, L**H**L, R**H**L, WG (2), KCL, YLL
^I^Germin-like proteins (6^T^)	214 (5^c^ + 1^f^) (181–225)	9 (5–16)	0.0403 (0.0231–0.0751)	LY (1)^4^ (2)^2^, P**H**Q, P**H**T (1)^3^, KP (1)^2^ (2)^2^, WEN, FC (1)^3^ (2), EL (1)^3^, **H**I**H**, P**H**I, KVI (1)^2^, LK (1)^4^, YLL, **H**V**H**, P**H**V, P**H**K, VKL, LWV, LW, L**H** (2), AY, YQY, L**H**T, L**H**Y, PAGY, VY, TDY, LLPF, MM, LTC
^I,II^Enzymes + other2 (18^T^)	875 (16^c^) (571–990)	58 (42–88)	0.0674 (0.0446–0.0892)	ADGF (1)^7^, A**H** (1)^2^ (2)^7^ (3)^5^, A**HH**, AW (1)^9^ (2)^4^, AY (1)^6^ (2)^4^ (3)^3^ (4), AYY (1)^2^, CGA (1)^2^, CME (1)^2^, D**H**G, EL (1)^2^ (2)^3^ (3)^4^ (4)^3^ (5)^2^ (6), EAK (1)^2^, ECA (1)^2^, FC (1)^9^ (2)^4^, FKK, GAA (1) (2)^2^, GA**H**, GPP, G**HH** (1)^2^, GGE (1)^2^, GYY (2)^2^, GGRP (1)^5^, GEC, GTW, GAWA, **HH** (1)^7^ (2)^2^ (3), **H**L (1) (2)^4^ (3)^6^ (4) (5) (6) (8), **HHH** (1)^2^, **HH**P (1)^2^, **H**L**H** (1)^3^, **HH**W (1)^2^, **H**V**H** (1)^2^, **HH**Y, I**HH**, IY (1)^6^ (2)^4^ (3)^4^, IR (1)^2^ (2)^4^ (3)^3^ (4)^2^ (5) (7) (8), IKL (1)^6^, IKK (1)^3^, IQY (1)^2^, IYY (1)^2^, KCL (1)^3^, KD (1) (2)^4^ (3)^4^ (4)^4^ (6), KP (1)^5^ (2)^6^ (3) (4) (5), KYL (1)^3^, KVI (1)^2^, L**H**A (1)^2^, L**H**D (2)^2^, L**H**E (1)^2^, LAC (1)^2^, LDY, LFC, L**H**V, L**H** (1) (2)^5^ (3) (4)^2^ (5)^2^ (6) (7), L**HH** (1)^2^, L**H**F (1)^2^, L**H**I (1)^2^, L**H**L (1)^7^ (2), L**H**S (1)^2^, LLR (1)^2^, LK (2) (3)^3^ (5)^5^ (6)^3^ (7) (8)^2^, LW (1)^3^ (2)^2^, LWA (1)^2^, L**H**T (1)^2^, LWG (1)^3^, LY (1)^5^ (2)^4^ (3)^3^ (4)^3^, L**H**Q, LKP (1)^6^, L**H**G (1)^4^ (2), LARL, LWK, LWD, L**H**K (1)^3^, L**H**M (1)^3^, MM (1)^7^, MY (1)^3^ (2)^8^, P**H**D, P**H**E (1)^2^, P**H**G (1)^3^, P**H**N, P**H**L (1)^2^, PWL, P**H**V, PWE, PWQ (1)^4^, PWV (1)^2^, PW (1)^9^, PWS, R**H**F, R**H**R (1)^2^, R**H**S, RW (1)^6^ (2)^2^ (3), RWW (1)^2^, R**H**Q (1)^2^, RWA (1)^3^, RWG (1)^2^, RWI, R**H**L (1)^2^, R**H**K, R**H**D (1)^8^, RWE, R**H**G (1)^3^, SWN (1)^2^, S**HH** (1)^2^, SYY (1)^2^, TY (1)^3^ (2)^4^ (3)^5^ (5), TW (1)^3^ (2), T**HH** (1)^2^, WG (1)^6^ (2) (3)^2^, WPL, V**HH**, VKL (1)^4^ (2), VW (1)^6^, VKV (1)^3^, VKP (1)^8^ (2), VVKL (1)^2^, YVL (1)^2^, VY (1)^5^ (2) (3)^6^ (4)^2^, YYL (2)^2^, YLL (1)^3^, Q**HH**, QYY (1)^2^, YYA (1)^2^, YYD, YYQ (1)^2^, YKY (1)^2^, YRY (1)^2^, YYG, YDD, YLY (2)^2^, Y**HH**, YYR
^I^Actins + other3 (23^T^)	355 (20^c^ + 2^f^) (202–514)	26 (15–40)	0.0734 (0.0453–0.1217)	A**H**K, A**H** (1)^5^ (2)^4^, ADF, AGDDAPR (1)^3^, AW (1)^7^ (2), AY (1)^6^ (2)^5^ (3)^2^ (5), CGA, C**H**I (1)^2^, D**HH** (1)^2^, DYK (1)^2^, EL (1)^2^ (2)^8^ (3)^6^ (4) (5)^2^ (6), ELLI, EAK, FC (1)^3^ (2), FYY (1)^4^, GAA (1)^5^ (2)^4^, GA**H** (1)^2^, CLV (1)^2^, **HH** (1)^9^ (7)^2^, **H**L (1)^8^ (2)^3^ (3)^2^, **HHH** (5)^2^, **HH**P (1)^2^, **H**L**H**, I**HH**, IY (1)^9^ (2)^2^ (6), IR (1)^11^ (2)^5^, IRW, IKL, KCL, KD (1)^7^ (2)^5^, KP (1)^5^ (2) (4)^2^, KKY, KVI, LAC (1)^3^, LDY (1)^4^, LFC (2), L**H**F (1)^2^ (3)^2^, L**H**V, L**H** (1)^6^ (2)^3^ (3)^3^, L**HH**, L**H**I, L**H**L (1)^4^, L**H**S (1)^3^, L**H**Y, LLR (1)^3^ (2)^2^, LK (1)^10^ (2)^2^, LW (1)^9^ (2) (5), LWA (2), L**H**T, LWI (1) (2), LW**H**, LWG (1)^3^, LWR, LWY, LY (1)^9^ (2)^6^ (3)^2^, LVSK, MM (1)^6^, MY (1)^6^ (2), PEL (1)^3^, P**H**A (1)^3^, P**H**E, P**H**G (1)^2^, P**H**N (1)^2^, P**H**M, PW (1)^6^, PWA, PWG, PWS, PWI (1)^3^, R**H**N (1)^2^, R**H**I, R**H**S (1)^3^, R**H**Y, R**H**T (1)^3^, RW (1)^7^ (2), RWL, SYY, TY (1)^13^, TDY, TERGY (1)^4^, TW (1)^6^, TFE, WDDMEK (1)^4^, WG (1)^6^ (2)^3^, W**HH** (1)^4^, WY (1)^6^, VAPEE**H**PV (1)^2^, V**HH**, VKL, VW (1)^5^ (2)^5^, VY (1)^8^ (2)^4^, VYV, YKY, YMY (1)^2^, YVL, YVGD (1)^4^, YY**H** (1)^2^, YYM, YYN, YYL, YQL, Y**HH** (1)^2^, YAY, QCL (1)^2^, Q**HH**
^I,II^Other4 (4^T^)	330 (2^c^) (318–342)	16 (13–18)	0.0468 (0.0409–0.0526)	L**H**, A**H** (2), YYD, SYY, L**H**G, PWA, R**H**E, R**H**Q, MY, GGE (2), KD (1) (2), PW, VY, LLR, LAC, EL (3), WY, EAK, IR (2), LK (3), ALSAF, GAA

n^T^—the number of total proteins including all protein fragments available from the BIOPEP-UWM database (access on August/September 2019 and from the 1st to the 10th December 2019. The data were updated as of 10 December 2019), n—the number of analysed proteins including selected fragments, ^c,f^—protein sequence status (^c^-complete, ^f^-a protein fragment), x—a mean value, *NAAR—the number of amino acid residues found in the analysed protein or protein fragment, **NAFS—the number of potential sequence fragments with an antioxidant activity, ***A—the A parameter (the frequency of bioactive fragment occurrence in a protein sequence) (defined in the “Materials and Methods” section of the study), ****AAS—amino acid sequence presented in the form of a single-letter amino acid code: A—Alanine, R—Arginine, N—Asparagine, D—Aspartic acid, C—Cysteine, Q—Glutamine, E—Glutamic acid, G—Glycine, H—Histidine, I—Isoleucine, L—Leucine, K—Lysine, M—Methionine, F—Phenylalanine, P—Proline, S—Serine, T—Threonine, W—Tryptophan, Y—Tyrosine, V—Valine. The number of bioactive fragments with a particular amino acid sequence found in a single analysed protein is provided in brackets. The number of proteins in which the particular bioactive fragment(s) was (were) found is provided in the superscript. The histidine residues found in the bioactive sequences are highlighted in bold, *****—globulins results have been marked, ******—expansin-B1 results has been marked. Other (1-4)—based on the number of amino acid residues found in the analysed protein or protein fragment and the A parameters were classified proteins.

**Table 3:** Characteristics of barley proteins antioxidant activity (^I^*Hordeum vulgare).*

Protein Groups (n^T^)	NAAR* x (n^c, f^) (Range)	NAFS** x (Range)	A*** x (Range)	AAS**** (Antioxidant Activity)
^I^Hordeins (16^T^)	334 (6^c^ + 4^f^) (105–475)	13 (7–44)	0.0344 (0.0190–0.0614)	LY (1)^3^ (2)^3^, P**H**Q (1)^8^ (2) (15), PWQ (1)^2^ (3), PW (1)^2^ (2)^2^ (3), IR (1)^4^, LLPF (1)^2^, QCL, EL (1)^3^ (2)^2^, PYPQ (1)^2^ (2) (3)^2^, P**H**I, LK (1)^2^, KP (1)^3^, LPM (1)^2^, LWS, LW (1)^2^, R**H**E (1)^3^, IQY, VY (1)^3^, QKPFPQQPPF, PQIPEQF, LRTLPM, SVNVPL (1)^2^, MM, A**H**, GYY (4)^2^, PWS (2)^2^, LQPGQGQQ (1)^2^, LQPGQGQQG (1)^2^, TY, WG (1)^2^, ACQ (1)^2^, L**H** (2), L**HH**, **HH**, AY, L**H**Q, WYY, WY, YYG, LYY (2)
^I^Globulin***** + hordothionins + other1 (10^T^)	197 (6^c^ + 2^f^) (127–499)	13 (2–45)	0.0587 (0.0157–0.0902)	KP (1)^2^ (2)***** (2) (3), WG*****, LAC*****, **H**L (1) (3), IKK (1)^2^, IY (1)^2^, KD (1) (2), IR (1)^2^ (2), TY (1) (2) (3), VW, AY (2) (4), EL (1)^2^ (2), EAK, KAI (2), LK (1)^3^ (2)^2^, VKP, GAA (1) (2), **HH**P, L**HH**, L**H** (2), LL**HH**, **HH**, ADF, LY (1)^4^, A**H**, YEY, YGY, L**H**E, RWG (3), IRW (2), RW (4), WG (2) (5), FC (1)^2^ (2), VY (2), YVE, PWQ, PW, YAY, L**H**N, L**H**S
^I^Tubulin chains (4^T^)	450 (4^c^) (447–451)	16 (15–20)	0.0362 (0.0333–0.0447)	AY (1) (2)^2^, LY (1)^4^, IY (1)^2^ (2), A**H**, EL (1)^3^ (2), WY (1)^4^, R**H**G (1)^4^, MY (2)^3^, KD (2)^3^, TY (1)^2^ (2), MM (1)^3^ (2), IR (1)^3^, VY (1)^3^, L**H** (3), **H**L, YYN, VYY, L**H**F, L**H**I, L**H**W, PEL, LK
^I^Thaumatin-like proteins (4^T^)	214 (4^c^) (171–233)	10 (8–13)	0.0478 (0.0429–0.0575)	AY (1)^4^, EL (1)^3^, YSY (1)^3^, KVI, KD (1) (2) (3), TDY, TW (1)^2^, VW (1) (2)^3^, FC (1)^2^, LK (1)^2^, TY (1)^2^, LAC (1)^2^, VY, YLL, GGE, RYQ, WG, LPM
^I^Hordoindolines (14^T^)	136 (14^f^) (103–142)	10 (4–14)	0.0773 (0.0282–0.1165)	L**H** (1)^10^, GYY (1)^8^, EL (1)^10^, YYW (1)^8^, L**H**D (1)^10^, RWW (1)^8^ (2)^2^, VKV (1)^10^, KVI (1)^10^, KD (1)^10^ (2)^4^, RW (1)^8^ (2)^2^, TW (1)^6^ (2)^8^, YLL (1)^10^, IR (1)^4^, QYP
^I^Enzymes + other2 (11^T^)	162 (6^c^ + 3^f^) (103–245)	8 (4–16)	0.0530 (0.0272–0.1165)	EL (1)^4^ (2)^4^, YYQ, SYY, MY, KD (1)^5^ (2), VY (1)^3^, CGA, VKL, PW (1)^3^, MWC, YCY, LK (1)^3^, PWT, GPP, FC (2), YVE, LY (1)^2^, LWI, YVL, LW, FKK, AY, A**H** (1)^3^, YYV (1)^2^, NYY (1)^2^, R**H**V (1)^2^, YVL (1)^2^, IR (1)^2^ (2), AW, P**H**N, TY (1) (2), L**H**, L**H**I, **H**E**H**, **H**R**H**, PWE, R**H**R, RW, KP, IY

n^T^—the number of total proteins including all protein fragments available from the BIOPEP-UWM database (access on August/September 2019 and from the 1st to the 10th December 2019. The data were updated as of 10 December 2019), n—the number of analysed proteins including selected fragments, ^c,f^—protein sequence status (^c^-complete, ^f^-a protein fragment), x—a mean value, *NAAR—the number of amino acid residues found in the analysed protein or protein fragment, **NAFS—the number of potential sequence fragments with an antioxidant activity, ***A—the A parameter (the frequency of bioactive fragment occurrence in a protein sequence) (defined in the “Materials and Methods” section of the study), ****AAS—amino acid sequence presented in the form of a single-letter amino acid code: A—Alanine, R—Arginine, N—Asparagine, D—Aspartic acid, C—Cysteine, Q—Glutamine, E—Glutamic acid, G—Glycine, H—Histidine, I—Isoleucine, L—Leucine, K—Lysine, M—Methionine, F—Phenylalanine, P—Proline, S—Serine, T—Threonine, W—Tryptophan, Y—Tyrosine, V—Valine. The number of bioactive fragments with a particular amino acid sequence found in a single analysed protein is provided in brackets. The number of proteins in which the particular bioactive fragment(s) was (were) found is provided in the superscript. The histidine residues found in the bioactive sequences are highlighted in bold, *****—globulin results has been marked. Other1,2—based on the number of amino acid residues found in the analysed protein or protein fragment and the A parameters were classified proteins.

**Table 4:** Characteristics of oat proteins antioxidant activity (^I^*Avena sativa)*.

Protein Groups (n^T^)	NAAR* x (n^c, f^) (Range)	NAFS** x (Range)	A*** x (Range)	AAS**** (Antioxidant Activity)
^I^12S + 11S globulins (5^T^)	481 (4^c^ + 1^f^) (313–551)	20 (13–22)	0.0421 (0.0380–0.0466)	**H**L (1)^2^ (2), **HH** (1)^2^, V**HH** (1)^2^, AY (1)^2^ (2)^3^, LY (1)^5^, A**H** (1)^5^, PWQ (1)^4^, KD (1)^4^, PW (1)^4^, IR (1) (2)^4^, LKP (1)^5^, LK (3)^3^ (4)^2^, KP (1)^5^, TY (1)^5^, VY (2)^4^, FC (1)^2^ (2)^2^, VYV (1)^2^, L**H**, IY (2)^2^, L**H**R
^I^Avenins (9^T^)	210 (4^c^) (182–222)	4 (2–8)	0.0166 (0.0090–0.0374)	VY (1)^3^, VYV (1)^3^, AY, EL (2), PW, LK (2), TW (2)
^I^Thaumatin-like pathogenesis- related proteins (4^T^)	169 (4^c^)	8 (8–9)	0.0488 (0.0473–0.0533)	**HH**P (1)^3^, Y**HH** (1)^3^, **HH** (1)^3^, AY (1)^4^, LK (1)^3^, VW (1)^4^, WG (1)^4^, FC (1)^4^, DYY, YYD, GGE, VY, TW
^I^Avenoindolines + other (5^T^)	148 (2^c^)	10 (8–11)	0.0642 (0.0541–0.0743)	GYY (1)^2^, WY, YYW (1)^2^, RWW (1)^2^, KD (1)^2^, RW (1)^2^, TW (1)^2^, IR, EL, EAK, KVI, GPP, YLL

n^T^—the number of total proteins including all protein fragments available from the BIOPEP-UWM database (access on August/September 2019 and from the 1st to the 10th December 2019. The data were updated as of 10 December 2019), n—the number of analysed proteins including selected fragments, ^c, f^—protein sequence status (^c^ -complete, ^f^-a protein fragment), x—a mean value, *NAAR—the number of amino acid residues found in the analysed protein or protein fragment, **NAFS—the number of potential sequence fragments with an antioxidant activity, ***A—the A parameter (the frequency of bioactive fragment occurrence in a protein sequence) (defined in the “Materials and Methods” section of the study), ****AAS—amino acid sequence presented in the form of a single-letter amino acid code: A—Alanine, R—Arginine, N—Asparagine, D—Aspartic acid, C—Cysteine, Q—Glutamine, E—Glutamic acid, G—Glycine, H—Histidine, I—Isoleucine, L—Leucine, K—Lysine, M—Methionine, F—Phenylalanine, P—Proline, S—Serine, T—Threonine, W—Tryptophan, Y—Tyrosine, V—Valine. The number of bioactive fragments with a particular amino acid sequence found in a single analysed protein is provided in brackets. The number of proteins in which the particular bioactive fragment(s) was (were) found is provided in the superscript. The histidine residues found in the bioactive sequences are highlighted in bold. Other—based on the number of amino acid residues found in the analysed protein or protein fragment and the A parameters were classified proteins.

**Table 5:** Characteristics of buckwheat (^I^*Fagopyrum esculentum,* ^II^*Fagopyrum gracilipes,* ^III^*Fagopyrum tataricum*), rye (^IV^*Secale cereale*) and sorghum (^V^*Sorghum vulgare*) proteins antioxidant activity.

Protein Groups (n^T^)	NAAR* x (n^c, f^) (Range)	NAFS** x (Range)	A*** x (Range)	AAS**** (Antioxidant Activity)
^I^13S globulins + ^II^seed storage protein (6^T^, 5^I^ + 1^II^)	408 (5^c^ + 1^f^) (191–565)	18 (9–24)	0.0460 (0.0353–0.0628)	**HH** (1)^3^, LY (2)^3^ (3)^2^, A**H** (1)^6^, EL (1)^3^ (2)^3^, F**HH**, P**H**R, PWR, RWN (1)^4^, PW (1)^3^, RW (1)^4^, IR (1)^4^, LK (1) (2) (3) (5)^3^, FC (1)^5^, P**HH** (1)^2^, L**H** (1)^2^, AY (1)^3^, L**H**E, PWQ (1)^2^, R**H**S, TY (1)^2^, LW, IKK, ADF, IY, P**H**W, **H**L, **H**E**H**, YYS, SYY, L**H**G, R**H**N, YVL, WPL, VY, AW, VW, VYV, YVE, KP
^I^Legumin-like 13S + legumin-type protein (2^T^)	232 (2^f^) (160–303)	7	0.0334 (0.0231–0.0437)	P**HH**, **HH** (1)^2^, PWQ, R**H**S, PW (1)^2^, IR (1)^2^, LW, LY, F**HH**, P**H**R, PWR
^I^Vicilin-like protein (1^T^)	140 (1^c^)	4	0.0286	EL (2), P**H**I, EAK
^I,III^Allergenic protein + other1 (4^T^, 3^I^ + 1^III^)	188 (3^c^ + 1^f^) (133–252)	8 (6–11)	0.0420 (0.0238–0.0578)	EL (1)^2^, MY, KD (1) (2), LK (1) (3), VY, TW, MM (3), EQC, AY (1)^2^, A**H** (1)^2^, LY (2), **H**YY, YY**H**, RWR, RW (1)^2^, IR (2), RWN, FC
^IV^Omega-secalins***** + other2 (14^T^)	357***** (1^c^)	1*****	0.0028*****	EL*****
^IV^Glutenin,^,^ HMW subunits (2^T^)	734 (2^c^) (713–754)	31 (25–36)	0.0414 (0.0351–0.0477)	GYY (9) (18), EL (1) (2), EYY (2), RYY, YYI (1)^2^, YYL (2), YYS, SYY (1)^2^, LWG, P**H**Q, LK (3), KP (1)^2^, LW, WG (1)^2^, IY, YYQ, P**H**Y, PWS, R**H**V, LQPGQGQQ (2), LQPGQGQQG (2), PW, IR
^V^Kafirins (3^T^)	268 (3^c^) (267–269)	14 (13–16)	0.0523 (0.0483–0.0599)	L**H** (1)^3^, **H**L (1) (2)^2^, AY (5) (6) (8), A**H** (1)^3^, L**H**A (1)^2^, P**H**S (1)^2^, TY (1)^2^, L**H**T, LLR, LLPF (2), LPM, IR

n^T^—the number of total proteins including all protein fragments available from the BIOPEP-UWM database (access on August/September 2019 and from the 1st to the 10th December 2019. The data were updated as of 10 December 2019), n—the number of analysed proteins including selected fragments, ^c, f^—protein sequence status (^c^-complete, ^f^-a protein fragment), x—a mean value, *NAAR—the number of amino acid residues found in the analysed protein or protein fragment, **NAFS—the number of potential sequence fragments with an antioxidant activity, ***A—the A parameter (the frequency of bioactive fragment occurrence in a protein sequence) (defined in the “Materials and Methods” section of the study), ****AAS—amino acid sequence presented in the form of a single-letter amino acid code: A—Alanine, R—Arginine, N—Asparagine, D—Aspartic acid, C—Cysteine, Q—Glutamine, E—Glutamic acid, G—Glycine, H—Histidine, I—Isoleucine, L—Leucine, K—Lysine, M—Methionine, F—Phenylalanine, P—Proline, S—Serine, T—Threonine, W—Tryptophan, Y—Tyrosine, V—Valine. The number of bioactive fragments with a particular amino acid sequence found in a single analysed protein is provided in brackets. The number of proteins in which the particular bioactive fragment(s) was (were) found is provided in the superscript. The histidine residues found in the bioactive sequences are highlighted in bold, *****—ω-secalins results have been marked. Other1,2—based on the number of amino acid residues found in the analysed protein or protein fragment and the A parameters were classified proteins. HMW—high molecular weight.

**Table 6:** The bioparameters characterising the in silico hydrolysis of wheat proteins (^I^Triticum aestivum, ^II^Triticum aestivum subsp. Spelta, ^III^Triticum aestivum spp. sphaeroccoum, ^IV^Triticum aestivum spp. compactum, ^V^Triticum aestivum spp. tibeticum, ^VI^Triticum turgidum subsp. durum) and the molecular characterisation of the obtained antioxidant peptides.

Protein Groups (n^T^)	Ficin (EC 3.4.22.3)			Stem Bromelain (EC 3.4.22.32)			Pepsin (pH > 2) (EC 3.4.23.1)		
	DH_t_ (%)* x (n^c, f^) Range	A_E_** x Range	AAS- AP*****	DH_t_ (%)* x (n) Range	A_E_** x Range	AAS- AP*****	DH_t_ (%)* x (n) Range	A_E_** x Range	AAS- AP*****
	NAP**** x Range	W*** x *Range*		NAP**** x Range	W*** x *Range*		NAP**** x Range	W*** x *Range*	
^I^Alpha- gliadins (21^T^, 20^I^ + 1^II^)	27.87 (14^c^ + 2^f^) 24.92–30.07	0.0044 0.0031–0.0105	A**H** (1)^2^, EL (1)^2^, TY (1)^16^	31.85 (14^c^ + 2^f^) 29.07–35.07	0.0042 0.0031–0.0073	EL (1)^2^, YQL (1)^16^, IR	66.35 (16^c^ + 2^f^) 63.26–68.77	0.0064 0.0031–0.0077	**H**L (1)^12^, VY (1)^18^, P**H**Q (1)^2^
	1 1–3	0.0907 0.0532–0.2008		1 1–2	0.0853 0.0658–0.1426		2 1–2	0.1285 0.0532–0.154	
^I^Alpha/beta- gliadins (12^T^)	27.02 (9^c^ + 1^f^) 24.84–29.19	0.0036 0.0031–0.0054	TY (1)^10^	33.01 (8^c^ + 1^f^) 32.35–35.09	0.0036 0.0031–0.0054	YQL (1)^9^	67.04 (10^c^ + 1^f^) 64.75–68.87	0.0047 0.0031–0.0108	**H**L (1)^3^, VY (1)^10^, P**H**Q
	1	0.0807 0.0521–0.1003		1	0.0811 0.0521–0.1003		1 1–2	0.1005 0.0521–0.1674	
^I^Gamma- gliadins (32^T^)	27.33 (3^c^ + 2^f^) 24.58–30.03	0.0041 0.0033–0.0049	AY (1)^2^, IR (1)^3^	33.64 (21^c^ + 6^f^) 28.51–49.91	0.0045 0.0030–0.0072	**H**L, IR (1)^21^ (2)^6^	64.44 (20^c^ + 8^f^) 62.63–67.08	0.0090 0.0035–0.0119	**H**L, IY (1)^2^, P**H**Q (1)^6^ (2)^19^, VY (1)^22^
	1	0.1231 0.0831–0.1667		1 1–2	0.1452 0.0831–0.2521		2 1–3	0.2667 0.1655–0.6694	
^III,IV^Gamma- gliadins (6^T^, 4^III^ + 2^IV^)	-	-	-	29.90 (6^f^) 29.28–31.21	0.0038 0.0035–0.0041	IR (1)^6^	64.83 (6^f^) 63.95–65.60	0.0114 0.0106–0.0123	P**H**Q (2)^6^, VY (1)^6^
	-	-	-	1	0.0832 0.0824–0.0842		3	0.2501 0.2494–0.2505	
^I^Omega- gliadins (3^T^)	24.01 (1^c^)	0.0107	EL (2), PEL	26.16 (1^c^)	0.0071	EL, PEL	65.23 (1^c^)	0.0036	PWQ
	3	0.3741		2	0.2483		1	0.1259	
^I^Glutenin, HMW subunits (12^T^)	38.82 (12^c^) 38.07–41.03	0.0043 0.0031–0.0065	EL (1)^6^ (2)^6^, WG (1)^12^, PWS (1)^5^, WY, IY	41.02 (12^c^) 39.01–43.12	0.0058 0.0031–0.0087	EL (1)^6^ (2)^6^, YYL (1) (2)^5^, YYS (1)^5^, WG (1)^12^, PWS (1)^5^	75.12 (12^c^) 73.70–76.72	0.0028 0.0021–0.0042	PWY (1)^4^, WG (1)^12^, WY (1)^4^, P**H**Y, R**H**Y, **H**L, IY
	3 2–4	0.1248 0.0701–0.1860		4 2–6	0.1561 0.1197–0.1860		2 1–3	0.0827 0.0462–0.127	
^I^Glutenin, HMW subunits (21^T^)	39.15 (1^c^ + 14^f^) 35.83–41.98	0.0156 0.0084–0.0252	EL (1)^7^ (2)^8^, WG (1)^13^, PWS (1)^4^	50.23 (1^c^ + 16^f^) 42.92–58.82	0.0147 0.0045–0.0252	EL (1)^6^ (2)^8^, WG (1)^13^, PWS (1)^4^, YYL (1)^4^, YYS	69.40 (1^c^ + 12^f^) 66.37–72.17	0.0065 0.0042–0.0110	WG (1)^13^, WY (1)^2^
	3 2–3	0.3213 0.1250–0.5000		3 1–4	0.2976 0.0828–0.5000		1 1–2	0.1229 0.0625–0.2222	
^I,V^Glutenin, LMW subunits (57^T^, 54^I^ + 2^V^)	29.03 (38^c^ + 5^f^) 26.26–31.31	0.0054 0.0029–0.0094	A**H** (1)^10^, IR (1)^12^, VY (1)^17^ (2)^2^, AY (1)^27^, WY (1)^4^, EL (1)^2^	38.18 (42^c^ + 13^f^) 28.46–43.88	0.0038 0.0026–0.0072	IR (1)^55^, **H**L (1)^8^, EL (1)^2^	67.41 (42^c^ + 13^f^) 64.36–70.62	0.0085 0.0052–0.0142	IY (1)^24^, VY (1)^29^ (2)^6^, P**H**Q (1)^41^ (2)^9^, **H**L (1)^14^, P**H**K, WY (1)^4^, PWQ (1)^4^, P**H**L
	2 1–3	0.1930 0.0768–0.4264		1 1–2	0.1346 0.0778–0.2229		3 2–4	0.3129 0.1535–0.4437	
^I^Enzyme inhibitors (10^T^)	39.53 (7^c^) 34.51–43.06	0.0119 0.006–0.0242	EL (1)^3^, IY, AY (1)^2^, A**H**, MY (1)^2^, PWS, VY	54.16 (5^c^) 48.59–58.33	0.011 0.0069–0.0165	**H**L, EL, PWS, YYL (1)^2^, PEL (1)^2^	59.97 (3^c^) 59.02–60.48	0.0070 0.0060–0.0081	**H**L, IY, VY
	2 1–3	0.2213 0.1261–0.3340		1 1–2	0.2235 0.1660–0.2850		1	0.1529 0.1261–0.1667	
^I^Thaumatin- like proteins (3^T^)	-	-	-	52.94 (1^c^)	0.0065	WG	-	-	-
	-	-		1	0.1252		-	-	
^I^Other (13^T^, 12^I^ + 1^VI^)	43.84 (4^c^ + 1^f^) 36.11–48.72	0.0055 0.0040–0.0085	TY, WG (1)^3^, EL	56.75 (2^c^) 55.56–57.94	0.0063 0.0040–0.0085	EL, PWV	68.07 (4^c^) 65.41–70.24	0.0094 0.0079–0.0108	WG (2)^3^, IY, VPW
	1	0.1013 0.0633–0.1433		1	0.1033 0.0633–0.1433		2	0.1811 0.1250–0.2512	

n^T^—the number of total proteins including all protein fragments available from the BIOPEP-UWM database (access on August/September 2019 and from the 1st to the 10th December 2019. The data were updated as of 10 December 2019), n—the number of analysed proteins including selected fragments, ^c, f^—protein sequence status (^c—^complete, ^f^-a protein fragment), x—a mean value, *DH_t_—the theoretical degree of hydrolysis. Since antioxidant peptides were not always found among the obtained products resulting from the conducted proteolysis simulation to calculate the average DH_t_ values, the results of these proteolysis simulations which yielded antioxidant peptides were taken into account. **A_E_—the frequency of fragments with antioxidant activity released by a selected enzyme, ***W—the relative frequency of fragments with antioxidant activity released by a selected enzyme. The bioparameters (*DH_T_, **A_E_, ***W) which characterise the *in silico* hydrolysis process are explained in the “Materials and Methods” section of the paper. ****NAP—the number of antioxidant peptides, *****AAS-AP—the amino acid sequence of antioxidant peptides presented in the form of a single-letter amino acid code: A—Alanine, Q—Glutamine, E—Glutamic acid, G—Glycine, H—Histidine, I—Isoleucine, L—Leucine, M—Methionine, P—Proline, S—Serine, T—Threonine, W—Tryptophan, Y—Tyrosine, V—Valine. The number of bioactive peptides with a particular amino acid sequence found in a single analysed protein is provided in brackets. The number of proteins in which the particular bioactive peptide(s) was (were) found is provided in the superscript. The histidine residues found in the bioactive sequences are highlighted in bold. HMW—high molecular weight, LMW—low molecular weight.

**Table 7:** The bioparameters characterising the *in silico* hydrolysis of rice proteins (^I^*Oryza sativa,* ^II^*Oryza sativa* [japonica cultivar-group], ^III^*Oryza sativa subsp. japonica*) and the molecular characterisation of the obtained antioxidant peptides.

Protein Groups (n^T^)	Ficin (EC 3.4.22.3)			Stem Bromelain (EC 3.4.22.32)			Pepsin (pH > 2) (EC 3.4.23.1)		
	DH_t_ (%)* x (n^c, f^) Range	A_E_** x Range	AAS- AP*****	DH_t_ (%)* x (n^c, f^) Range	A_E_** x Range	AAS- AP*****	DH_t_ (%)* x (n^c, f^) Range	A_E_** x Range	AAS- AP*****
	NAP**** x Range	W*** x *Range*		NAP**** x Range	W*** x *Range*		NAP**** x Range	W*** x *Range*	
^I^Prolamins + globulins****** (11^T^)	40.62 (6^c^+ 1^f^******) 33.11-–52.59	0.0115 0.0067–0.0147	IY (1)^6^, EL (1) (2)******, VY (1)^3^	54.41 (4^c^+2^f^******) 50.97–57.80	0.0114 0.0057–0.0294	YYG (1)^3^, **H**L, WY, EL (1)****** (3)******	69.65 (7^c^ + 1^f^******) *67.10–73.15*	0.0118 0.0057–0.0256	IY (1)^6^, VY (1)****** (1) (2)^2^, **H**L, P**H**L
	2 1–2	0.2389 0.1675–0.3988		2 1–3	0.1972 0.1109–0.3634		2 1–4	0.2902 0.1109–0.5982	
^I^Glutelins (16^T^)	43.28 (7^c^) 42.63–44.06	0.0054 0.0039–0.0081	EL (1)^3^, VY (1)^4^ (2)^2^ (3), IY (1)^4^, AY	52.48 (7^c^) 50.90–54.81	0.0040 0.0020–0.0060	EL (1)^2^ (2)^2^, **H**L (1)^4^, IR (1)^4^	69.26 (7^c^) 68.37–71.74	0.0106 0.0078–0.0121	IY (1)^4^ (2)^2^, P**H**Y (1)^2^, VY (1) (2)^2^ (3) (4) (5)^2^, **HH**, **H**L, P**H**G, IR (1)^2^
	3 2–4	0.0979 0.0710–0.1434		2 1–3	0.0729 0.0364–0.1062		5 4–6	0.1916 0.1421–0.2169	
^I^Oryzains + ^II^expansin -B1******* (4^T^, 3^I^+1^II^)	42.04 (4^c^) 40.64–43.49	0.0083 0.0044–0.0112	EL (1)^2^, WG (1)******* (1) (2)^2^, MY, VY (1)******* (1), EAK*******	52.69 (4^c^) 47.74–59.28	0.0061 0.0037–0.0087	D**H**G (1)^2^, WG (1)******* (1) (2) (3), EL	68.67 (4^c^) 67.31–71.43	0.0054 0.0042–0.0075	R**H**D, WG (1)^3^, IY, VY (1)******* (1), WY*******
	3 2–4	0.1489 0.0876–0.2213		3 1–4	0.1176 0.0494–0.1733		2	0.0971 0.0876–0.1107	
^I^Lectin + oleosins (3^T^)	42.48 (1^c^)	0.0044	EL	50.89 (1^c^)	0.0088	EL (2)	68.58 (1^c^)	0.0044	WG
	1	0.1429		2	0.2857		1	0.1429	
^I^Glycine-rich proteins + other1 (9^T^)	58.30 (6^c^) 40.52–82.97	0.0105 0.0055–0.0148	A**H**, IR (1) (2), EL (1)^3^, TY (1)^3^	65.39 (5^c^) 53.62–82.42	0.0115 0.0055–0.0222	**HH**, EL (1)^2^, IR (1) (2), YYV, PWG, WG	72.44 (3^c^) 63.43–84.62	0.0118 0.0109–0.0130	**H**L, **HH**, PWG, VY, P**H**A, P**H**N
	2 1–2	0.3418 0.1259–0.6667		2 1–4	0.1998 0.1259–0.3744		2	0.2643 0.1434–0.4000	
^I,II^Allergenic proteins + homolog protein + ^II^profilin (13^T^)	41.92 (7^c^ + 4^f^) 39.23–45.77	0.0135 0.0070–0.0183	EL (1)^9^ (2), IY (1)^10^	55.63 (7^c^ + 4^f^) 39.44–59.82	0.0077 0.0060–0.0121	EL (1)^9^ (2), IR	62.02 (8^c^ + 4^f^) 56.36–73.08	0.0126 0.0088–0.0180	**H**L (1)^2^, P**H**D, IY (1)^11^, VY (1)^7^, R**H**E
	2 1–3	0.2934 0.0556–0.6655		1 1–2	0.1591 0.0556–0.3345		2 1–2	0.2520 0.1112–0.5000	
^I^Tubulin chains (5^T^)	41.68 (5^c^) 41.03–43.38	0.0058 0.0045–0.0089	AY, WY (1)^4^, MY (2), IR (1)^3^ (2), WG	51.30 (4^c^) 50.90–51.92	0.0061 0.0045–0.0107	EL (1)^3^, PEL (1)^3^, **H**L (2), IR (2), KCL	70.29 (5^c^) 68.15–71.33	0.0049 0.0044–0.0064	IY (2), **H**L (1)^4^, VY (1)^3^, WG (2)
	3 2–4	0.1509 0.0951–0.2862		3 2–5	0.1468 0.0951–0.2956		2 2–3	0.1220 0.0951–0.1768	
^I^Germin-like proteins (6^T^)	43.87 (1^c^)	0.0094	AY, EL	58.55 (3^c^) 54.71–66.04	0.0045 0.0044–0.0047	EL (1)^3^	69.02 (2^c^ + 1^f^) 66.07–72.64	0.0049 0.0044–0.0055	P**H**Q, P**H**K, VY
	2	0.1252		1	0.0926 0.0626–0.1236		1	0.1345 0.0626–0.1993	
^I,II^Enzymes + other2 (18^T^)	44.08 (16^c^) 41.44–48.18	0.0088 0.0032–0.0212	AY (1)^7^ (2)^2^ (3)^2^ A**H** (1)^9^ (2)^4^, EL (1)^8^ (2)^3^ (4)^2^, PWS, IR (1)^8^ (3) (5)^2^, TY (1)^8^ (2)^2^ (3), WG (1)^8^ (2), VY (1)^2^ (2)^4^, IY (1)^2^ (2)^3^ (3), EAK (1)^2^	54.74 (16^c^) 49.82–59.67	0.0078 0.0012–0.0127	**H**L (1)^4^ (2)^6^ (3)^2^ (4), EL (1)^3^ (2)^8^ (3), IR (1)^3^ (2)^2^ (3)^3^ (4)^3^ (5), D**H**G, WG (1)^9^ (2), YYG, YYA (1)^2^, YYL (1)^2^, PWL, IKL (1)^5^, KYL (1)^3^, WPL, PWV (1)^2^, YYR	68.29 (16^c^) 64.05–71.73	0.0105 0.0073–0.0139	**H**L (1) (2)^7^ (3)^6^ (4) (5), P**H**L (1)^2^, R**H**D, WG (1)^6^ (2)^2^ (3), IY (1)^6^ (2)^4^ (3)^4^, VY (1)^6^ (2) (3)^6^ (4)^2^, R**H**Q (1)^2^, PWL, R**H**K, SWN (1)^2^, WPL, **HH**Y, PWE, RWG, IR (1)^6^, V**HH**, RWE, R**H**G (1)^3^, PW (1)^2^, PWQ (1)^4^, RW
	8 3–21	0.1258 0.0663–0.2468		7 1–12	0.1195 0.0171–0.2313		9 6–12	0.1604 0.1038–0.2222	
^I^Actins + other3 (23^T^)	45.13 (19^c^ + 2^f^) 40.53–49.75	0.0101 0.0027–0.0175	EL (1)^11^ (2)^5^ (3) TDY, AY (1)^10^, IR (1)^3^ (2)^4^, A**H** (1)^3^, VY (1)^5^ (2)^2^, TY (1)^3^, WG (1)^4^, IY (1)^3^, AW, WY, MY (1)^3^, PEL, PWG	58.58 (19^c^ + 2^f^) 49.73–69.66	0.0093 0.0027–0.0217	P**H**A (1)^3^, IR (1)^5^ (2)^4^, **H**L (1)^6^ (2)^3^, EL (1)^6^ (2)^4^ (3)^3^ (4), YQL, WG (1)^7^ (2), PEL (1)^2^, YYL, PWG	68.42 (19^c^ + 2^f^) 62.82–70.91	0.0107 0.0030–0.0217	IY (1)^9^ (2) (6), P**H**A (1)^3^, VW (1)^2^ (2)^2^ VY (1)^8^ (2)^4^, **H**L (1)^5^ (2)^2^ (3), P**H**N (1)^2^, WG (1)^5^ (2), PW, WY (1)^2^, IR, P**H**E, P**H**G (1)^2^, R**H**N (1)^2^, RW (1)^2^, PWG, IRW, RWL, P**H**M
	4 1–8	0.1429 0.0293–0.3333		3 1–7	0.1279 0.0406–0.2382		4 1–10	0.1504 0.0662–0.2382	
^I,II^Other4 (4^T^)	41.32 (2^c^) 37.54–45.11	0.0061 0.0058–0.0063	AY, MY, EL, IR	56.15 (1^c^)	0.0063	EL, IR	71.30 (2^c^) 70.67–71.92	0.0030 0.0029–0.0031	VY, WY
	2	0.1322 0.1103–0.1540		2	0.1540		1	0.0655 0.0551–0.0758	

n^T^—the number of total proteins including all protein fragments available from the BIOPEP-UWM database (access on August/September 2019 and from the 1st to the 10th December 2019. The data were updated as of 10 December 2019), n—the number of analysed proteins including selected fragments, ^c, f^—protein sequence status (^c—^complete, ^f^-a protein fragment), x—a mean value, *DH_t_—the theoretical degree of hydrolysis. Since antioxidant peptides were not always found among the obtained products resulting from the conducted proteolysis simulation to calculate the average DH_t_ values, the results of these proteolysis simulations which yielded antioxidant peptides were taken into account. **A_E_—the frequency of fragments with antioxidant activity released by a selected enzyme, ***W—the relative frequency of fragments with antioxidant activity released by a selected enzyme. The bioparameters (*DH_T_, **A_E_, ***W) which characterise the *in silico* hydrolysis process are explained in the “Materials and Methods” section of the paper. ****NAP—the number of antioxidant peptides, *****AAS-AP—the amino acid sequence of antioxidant peptides presented in the form of a single-letter amino acid code: A—Alanine, R—Arginine, N—Asparagine, D—Aspartic acid, C—Cysteine, Q—Glutamine, E—Glutamic acid, G—Glycine, H—Histidine, I—Isoleucine, L—Leucine, K—Lysine, M—Methionine, F—Phenylalanine, P—Proline, S—Serine, T—Threonine, W—Tryptophan, Y—Tyrosine, V—Valine. The number of bioactive peptides with a particular amino acid sequence found in a single analysed protein is provided in brackets. The number of proteins in which the particular bioactive peptide(s) was (were) found is provided in the superscript. The histidine residues found in the bioactive sequences are highlighted in bold, ******—globulins results have been marked, *******—expansin-B1 results has been marked. Other (1-4)—based on the number of amino acid residues found in the analysed protein or protein fragment and the A parameters were classified proteins.

**Table 8:** The bioparameters characterising the *in silico* hydrolysis of barley proteins (^I^*Hordeum vulgare) *and the molecular characterisation of the obtained antioxidant peptides.

Protein Groups (n^T^)	Ficin (EC 3.4.22.3)			Stem Bromelain (EC 3.4.22.32)			Pepsin (pH>2) (EC 3.4.23.1)		
	DH_t_ (%)* x (n^c, f^) Range	A_E_** x Range	AAS- AP*****	DH_t_ (%)* x (n) Range	A_E_** x Range	AAS- AP*****	DH_t_ (%)* x (n) Range	A_E_** x Range	AAS- AP*****
	NAP**** x Range	W*** x Range		NAP**** x Range	W*** x Range		NAP**** x Range	W*** x Range	
^I^Hordeins (16^T^)	35.22 (3^c^ + 1^f^) 28.42–41.14	0.0041 0.0034–0.0053	EL (1)^3^, PQIPEQF, PWS (1)^2^, AY, WY	37.76 (5^c^ + 3^f^) 23.08–48.81	0.0048 0.0033–0.0095	IR (1)^4^, EL (1)^4^, PWS (1)^2^, YYG	64.51 (6^c^ + 3^f^) 60.58–69.79	0.0074 0.0013–0.0129	PWQ (1)^2^ (3), P**H**Q (1)^6^, RHE (1)^3^, VY (1)^3^, WY
	2 1–4	0.0994 0.0554–0.1264		1 1–3	0.1635 0.0688–0.5000		2 1–3	0.2534 0.0224–0.5022	
^I^Globulin****** + hordothionins + other1 (10^T^)	43.26 (3^c^ + 2^f^) 34.83–48.53	0.0188 0.0076–0.0274	IY, AY (2)^2^, EL (1) (2), ADF, A**H**, TY (2)^2^, VY, WG (1) (5), PWG	56.30 (3^c^ + 1^f^) 46.92–63.48	0.0113 0.0068–0.0153	**H**L, IR (1) (2), EL (1) (2), WG (3), PWG	69.29 (4^c^) 66.37–71.54	0.0171 0.0045–0.0382	WG (1)****** (1) (2), **H**L (1) (3), IY (1)^2^, VW, VY (2), PWG
	4 1–11	0.2383 0.0996–0.2661		3 1–6	0.1416 0.0993–0.2005		4 1–6	0.2713 0.1330–0.5007	
^I^Tubulin chains (4^T^)	42.22 (4^c^) 40.81–43.56	0.0106 0.0045–0.0133	AY (1) (2)^2^, WY (1)^4^, MY (2)^3^, IR (1)^3^, TY	52.82 (3^c^) 51.57–53.56	0.0030 0.0022–0.0045	IR (1)^2^, EL, PEL	69.36 (4^c^) 68.60–70.40	0.0044 0.0044–0.0045	IY (1)^2^ (2), VY (1)^3^, **H**L
	5 2–6	0.3082 0.1007–0.3994		1 1–2	0.0776 0.0661–0.1007		2	0.1243 0.1007–0.1321	
^I^Thaumatin- like proteins (4^T^)	41.91 (2^c^) 41.33–42.48	0.0044	EL (1)^2^	-	-	-	71.11 (1^c^)	0.0044	VY
	1	0.0882 0.0765–0.0998		-	-		1	0.0765	
^I^Hordoindolines (14^T^)	43.83 (5^f^) 42.55–46.08	0.0075 0.0070–0.0097	EL (1)^5^	51.63 (13^f^) 50.98–52.48	0.0089 0.0070–0.0141	EL (1)^4^, YYW (1)^8^, IR (1)^4^	-	-	-
	1	0.0768 0.0710–0.0833		1 1–2	0.1433 0.0765–0.2482		-	-	
^I^Enzymes + other2 (11^T^)	41.23 (3^c^ + 3^f^) 34.46–47.52	0.0149 0.0066–0.0291	EL (1)^3^ (2)^2^, MY, VY, AY, A**H** (1)^2^, TY	54.79 (5^c^ + 1^f^) 50.68–63.70	0.0091 0.0041–0.0194	EL (1)^5^, PWT, IR (1)^2^	63.48 (3^c^ + 3^f^) 60–70.49	0.0081 0.0066–0.0122	VY (1)^3^, PWT, P**H**N, IR, IY, PWE
	2 1–4	0.2782 0.1248–0.5000		1 1–2	0.1618 0.0628–0.2500		1 1–3	0.1646 0.0833–0.2006	

n^T^—the number of total proteins including all protein fragments available from the BIOPEP-UWM database (access on August/September 2019 and from the 1st to the 10th December 2019. The data were updated as of 10 December 2019), n—the number of analysed proteins including selected fragments, ^c, f^—protein sequence status (^c—^complete, ^f^-a protein fragment), x—a mean value, *DH_t_—the theoretical degree of hydrolysis. Since antioxidant peptides were not always found among the obtained products resulting from the conducted proteolysis simulation to calculate the average DH_t_ values, the results of these proteolysis simulations which yielded antioxidant peptides were taken into account. **A_E_—the frequency of fragments with antioxidant activity released by a selected enzyme, ***W—the relative frequency of fragments with antioxidant activity released by a selected enzyme. The bioparameters (*DH_T_, **A_E_, ***W) which characterise the *in silico* hydrolysis process are explained in the “Materials and Methods” section of the paper. ****NAP—the number of antioxidant peptides, *****AAS-AP—the amino acid sequence of antioxidant peptides presented in the form of a single-letter amino acid code: A—Alanine, R—Arginine, N—Asparagine, D—Aspartic acid, Q—Glutamine, E—Glutamic acid, G—Glycine, H—Histidine, I—Isoleucine, L—Leucine, M—Methionine, F—Phenylalanine, P—Proline, S—Serine, T—Threonine, W—Tryptophan, Y—Tyrosine, V—Valine. The number of bioactive peptides with a particular amino acid sequence found in a single analysed protein is provided in brackets. The number of proteins in which the particular bioactive peptide(s) was (were) found is provided in the superscript. The histidine residues found in the bioactive sequences are highlighted in bold, ******—globulin results has been marked. Other1,2—based on the number of amino acid residues found in the analysed protein or protein fragment and the A parameters were classified proteins.

**Table 9:** The bioparameters characterising the *in silico* hydrolysis of oat proteins (^I^*Avena sativa)* and the molecular characterisation of the obtained antioxidant peptides.

Protein Groups (n^T^)	Ficin (EC 3.4.22.3)			Stem Bromelain (EC 3.4.22.32)			Pepsin (pH>2) (EC 3.4.23.1)		
	DH_t_ (%)* x (n^c, f^) Range	A_E_** x Range	AAS- AP*****	DH_t_ (%)* x (n) Range	A_E_** x Range	AAS- AP*****	DH_t_ (%)* x (n) Range	A_E_** x Range	AAS- AP*****
	NAP**** x Range	W*** x *Range*		NAP**** x Range	W*** x *Range*		NAP**** x Range	W*** x *Range*	
^I^12S + 11S globulins (5^T^)	42.77 (4^c^) 42.40–43.33	0.0019 0.0018–0.0020	VY (1)^4^	50.59 (4^c^ + 1^f^) 49.04–52.18	0.0040 0.0019–0.0064	**H**L (1)^2^ (2), IR (1)^3^ (2)	71.13 (4^c^ + 1^f^) 69.55–71.82	0.0105 0.0064-0.0121	**H**L (1)^3^, VY (2)^4^, V**HH** (1)^2^, PWQ (1)^4^, IR (1)^5^, IY (2)^2^
	1	0.0452 0.0428–0.0500		2 1–2	0.0936 0.0500–0.1542		5 2–6	0.2497 0.1542–0.3000	
^I^Avenins (9^T^)	45.07 (1^c^)	0.0047	EL	52.11 (1^c^)	0.0047	EL	71.24 (3^c^) 69.61–72.60	0.0048 0.0045–0.0055	VY (1)^3^
	1	0.1257		1	0.1257		1	0.4982 0.4945–0.5000	
^I^Thaumatin-like pathogenesis- related proteins (4^T^)	-	-	-	-	-	-	68.45 (1^c^)	0.0059	VY
	-	-		-	-		1	0.1107	
^I^Avenoindolines + other (5^T^)	46.26 (1^c^)	0.0135	WY, IR	50.68 (2^c^) 50.34–51.02	0.0102 0.0068–0.0135	YYW (1)^2^, IR	65.99 (1^c^)	0.0068	WY
	2	0.2495		2 1–2	0.1705 0.0915–0.2495		1	0.1257	

n^T^—the number of total proteins including all protein fragments available from the BIOPEP-UWM database (access on August/September 2019 and from the 1st to the 10th December 2019. The data were updated as of 10 December 2019), n—the number of analysed proteins including selected fragments, ^c, f^—protein sequence status (^c—^complete, ^f^-a protein fragment), x—a mean value, *DH_t_—the theoretical degree of hydrolysis. Since antioxidant peptides were not always found among the obtained products resulting from the conducted proteolysis simulation to calculate the average DH_t_ values, the results of these proteolysis simulations which yielded antioxidant peptides were taken into account. **A_E_—the frequency of fragments with antioxidant activity released by a selected enzyme, ***W—the relative frequency of fragments with antioxidant activity released by a selected enzyme. The bioparameters (*DH_T_, **A_E_, ***W) which characterise the *in silico* hydrolysis process are explained in the “Materials and Methods” section of the paper. ****NAP—the number of antioxidant peptides, *****AAS-AP—the amino acid sequence of antioxidant peptides presented in the form of a single-letter amino acid code: A—Alanine, R—Arginine, Q—Glutamine, E—Glutamic acid, H—Histidine, I—Isoleucine, L—Leucine, P—Proline, W—Tryptophan, Y—Tyrosine, V—Valine. The number of bioactive peptides with a particular amino acid sequence found in a single analysed protein is provided in brackets. The number of proteins in which the particular bioactive peptide(s) was (were) found is provided in the superscript. The histidine residues found in the bioactive sequences are highlighted in bold. Other—based on the number of amino acid residues found in the analysed protein or protein fragment and the A parameters were classified proteins.

**Table 10:** The bioparameters characterising the *in silico* hydrolysis of buckwheat (^I^*Fagopyrum esculentum,* ^II^*Fagopyrum gracilipes,* ^III^*Fagopyrum tataricum*), rye (^IV^*Secale cereale*) and sorghum (^V^*Sorghum vulgare*) proteins and the molecular characterisation of the obtained antioxidant peptides.

Protein Groups (n^T^)	Ficin (EC 3.4.22.3)			Stem Bromelain (EC 3.4.22.32)			Pepsin (pH > 2) (EC 3.4.23.1)		
	DH_t_ (%)* x (n^c, f^) Range	A_E_** x Range	AAS- AP*****	DH_t_ (%)* x (n) Range	A_E_** x Range	AAS- AP*****	DH_t_ (%)* x (n) Range	A_E_** x Range	AAS- AP*****
	NAP**** x Range	W*** x Range		NAP**** x Range	W*** x Range		NAP**** x Range	W*** x Range	
^I^13S globulins + ^II^seed storage protein (6^T^, 5^I^ + 1^II^)	44.85 (4^c^ + 1^f^) 42.49–46.32	0.0042 0.0190–0.0066	PWR, IR (1)^4^, EL (1)^2^, IY, VY	55.48 (4^c^ + 1^f^) 54.87–57.37	0.0053 0.0037–0.0071	EL (1)^4^, P**H**R, PWR, IR (1)^3^, **H**L, YYS, WPL	68.15 (2^c^ + 1^f^) 64.80–71.50	0.0053 0.0019–0.0088	PWQ, **H**L, R**H**N, WPL, VY
	2 1–3	0.0969 0.0840–0.1358		2 1–4	0.1216 0.0828–0.2006		2 1–4	0.1157 0.0538–0.1811	
^I^Legumin- like 13S + legumin- type protein (2^T^)	44.69 (2^f^) 44.03–45.36	0.0065 0.0063–0.0066	IR (1)^2^, PWR	52.46 (2^f^) 50.94–53.97	0.0081 0.0063–0.0099	IR (1)^2^, P**H**R, PWR	-	-	-
	2 1–2	0.2150 0.1442–0.2857		2 1–3	0.2864 0.1442–0.4286		-	-	
^I^Vicilin-like protein (1^T^)	43.88 (1^c^)	0.0214	EL (2), EAK	51.80	0.0071	EL	-	-	-
	3	0.7483		1	0.2483		-	-	-
^I,III^Allergenic proteins + other (4^T^, 3^I^ + 1^III^)	44.82 (2^c^) 43.02–46.61	0.0885 0.0040–0.1730	EL, AY, A**H**, IR	54.12 (1^f^)	0.0051	EL	74.98 (2^c^) 72.67–77.29	0.0049 0.0040–0.0058	VY, RWR
	2 1–3	0.2337 0.1681–0.2993		1	0.0904		1	0.1342 0.1003–0.1681	
^IV^Omega- secalins + other (14^T^)	-	-	-	-	-	-	-	-	-
	-	-		-	-	-	-	-	
^IV^Glutenin,^,^ HMW subunits (2^T^)	38.22 (2^c^) 37.92-38.51	0.0048 0.0040–0.0056	IY, EL (1) (2), PWS, WG (1)^2^	40.00 (2^c^) 39.84–40.17	0.0061 0.0042–0.0080	EL (1) (2), PWS, WG (1)^2^, YYL (2), YYS	75.15 (2^c^) 75.00–75.30	0.0028 0.0013–0.0042	WG (1)^2^, IY, P**H**Y
	4 3–4	0.1217 0.0839–0.1595		5 3–6	0.1437 0.1197–0.1677		2 1–3	0.0735 0.0273–0.1197	
^V^Kafirins (3^T^)	32.13 (3^c^) 30.97–32.71	0.0050 0.0037–0.0075	A**H** (1)^3^, AY	53.00 (3^c^) 51.49–54.89	0.0050 0.0037–0.0075	**H**L (1)^3^, IR	77.37 (3^c^) 76.69–78.36	0.0062 0.0037–0.0075	**H**L (1) (2)^2^
	1 1–2	0.0926 0.0760–0.1252		1 1–2	0.0692 0.0618–0.1540		2 1–2	0.1075 0.0618–0.1540	

n^T^—the number of total proteins including all protein fragments available from the BIOPEP-UWM database (access on August/September 2019 and from the 1st to the 10th December 2019. The data were updated as of 10 December 2019), n—the number of analysed proteins including selected fragments, ^c, f^—protein sequence status (^c—^complete, ^f^-a protein fragment), x—a mean value, *DH_t_—the theoretical degree of hydrolysis. Since antioxidant peptides were not always found among the obtained products resulting from the conducted proteolysis simulation to calculate the average DH_t_ values, the results of these proteolysis simulations which yielded antioxidant peptides were taken into account. **A_E_—the frequency of fragments with antioxidant activity released by a selected enzyme, ***W—the relative frequency of fragments with antioxidant activity released by a selected enzyme. The bioparameters (*DH_T_, **A_E_, ***W) which characterise the *in silico* hydrolysis process are explained in the “Materials and Methods” section of the paper. ****NAP—the number of antioxidant peptides, *****AAS-AP—the amino acid sequence of antioxidant peptides presented in the form of a single-letter amino acid code: A—Alanine, R—Arginine, Q—Glutamine, E—Glutamic acid, H—Histidine, I—Isoleucine, L—Leucine, P—Proline, W—Tryptophan, Y—Tyrosine, V—Valine. The number of bioactive peptides with a particular amino acid sequence found in a single analysed protein is provided in brackets. The number of proteins in which the particular bioactive peptide(s) was (were) found is provided in the superscript. The histidine residues found in the bioactive sequences are highlighted in bold. Other—based on the number of amino acid residues found in the analysed protein or protein fragment and the A parameters were classified proteins. HMW—high molecular weight.
